# Global site-specific health impacts of fossil energy, steel mills, oil refineries and cement plants

**DOI:** 10.1038/s41598-023-38075-z

**Published:** 2023-08-22

**Authors:** Christopher Oberschelp, Stephan Pfister, Stefanie Hellweg

**Affiliations:** 1 ETH Zürich, Institute of Environmental Engineering, John-von-Neumann-Weg 9, 8093 Zurich, Switzerland; 2 National Centre of Competence in Research (NCCR) Catalysis, ETH Zürich, 8093 Zurich, Switzerland

**Keywords:** Environmental impact, Engineering

## Abstract

Climate change and particulate matter air pollution present major threats to human well-being by causing impacts on human health. Both are connected to key air pollutants such as carbon dioxide (CO$_\text {2}$), primary fine particulate matter (PM$_\text {2.5}$), sulfur dioxide (SO$_\text {2}$), nitrogen oxides (NO$_\text {x}$) and ammonia (NH$_\text {3}$), which are primarily emitted from energy-intensive industrial sectors. We present the first study to consistently link a broad range of emission measurements for these substances with site-specific technical data, emission models, and atmospheric fate and effect models to quantify health impacts caused by nearly all global fossil power plants, steel mills, oil refineries and cement plants. The resulting health impact patterns differ substantially from far less detailed earlier studies due to the high resolution of included data, highlighting in particular the key role of emission abatement at individual coal-consuming industrial sites in densely populated areas of Asia (Northern and North-Eastern India, Java in Indonesia, Eastern China), Western Europe (Germany, Belgium, Netherlands) as well as in the US. Of greatest health concern are the high SO$_\text {2}$ emissions in India, which stand out due to missing flue gas treatment and cause a particularly high share of local health impacts despite a limited number of emission sites. At the same time, the massive infrastructure and export capacity build-up in China in recent years is taking a substantial toll on regional and global health and requires more stringent regulation than in the rest of the world due to unfavorable environmental conditions and high population densities. The current phase-out of highly emitting industries in Europe is found not to have started with sites having the greatest health impacts. Our detailed site-specific emission and impact inventory is able to highlight more effective alternatives and to track future progress.

## Introduction

Industrial emissions, in particular from energy intensive sectors, have been identified as key sources of greenhouse gas (GHG) and particulate matter (PM) emissions, which both represent the two main causes of global pollution impacts^1–3^. The relevant substances associated with these impacts are mainly carbon dioxide (CO$_\text {2}$), methane (CH$_\text {4}$), primary particulate matter with a diameter below 2.5$\,\upmu$m (PM$_\text {2.5}$), sulfur dioxide (SO$_\text {2}$), nitrogen oxides (NO$_\text {x}$), ammonia (NH$_\text {3}$) and heavy metals. Numerous studies propose ways to mitigate global pollution impacts from industrial sources, but their scope is restricted by the availability of site-specific emission and pollutant fate data, which is commonly estimated by top-down approaches (e.g. with national instead of site-specific emission factors) combined with rough proxy data^4^. Thus, such studies cannot adequately account for highly location-specific pollutant impacts because of emission differences between industrial facilities (even of the same type), weather, atmospheric conditions, exposed population, or background pollution, while a lack of technical data per site also limits conclusions as to the effectiveness of specific improvement measures. Hence, realistic suggestions for improvements remain globally unavailable. We fill this gap by combining emission measurements with detailed global site-specific modeling of more than 125,000 power generating units, 3500 cement kilns, 1500 blast furnaces for primary steel production and 700 oil refineries (reaching nearly complete global coverage) and quantify pollution reduction potentials for GHG impacts and PM health impacts based on additional abatement measures. Thus, the present study is able to cover more than 90% of global stationary CO$_\text {2}$ emissions^5^.

### Global patterns of air pollution

**Figure 1:**
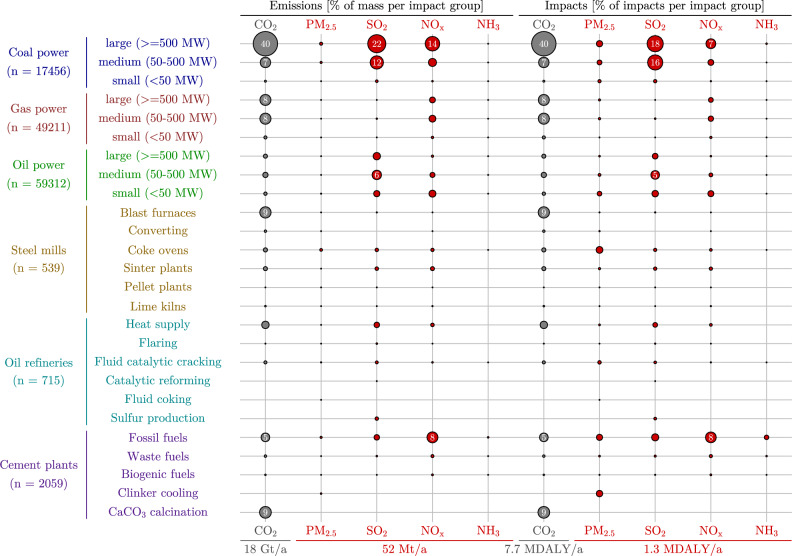
Sectoral emission and impact comparison in [%] contribution to totals in each category for 2016 (global warming-related in gray or PM human health impact-related in red). Total amounts for emissions and impacts given at the bottom of the figure. The split of health impacts between PM $_\text {2.5}$, *SO*$_\text {2}$, *NO*$_\text {x}$ *and NH*$_\text {3}$ is 18%, 53%, 27% and 2%, respectively. The full data is presented in supplementary table 24. Each steel mill may consist of several blast furnaces, while each cement plant may consist of several kilns.

Global patterns of air pollution are found to be closely linked with the use of coal in the various industry sectors. Not only does this type of fossil fuel have the highest CO$_\text {2}$ emission intensity among the three major types of fossil fuels (coal, gas, and oil), but it is also responsible for high levels of exposure to primary and secondary PM pollution. A direct comparison of the health impacts from global warming and PM shows that they are of a similar order of magnitude (Fig. 1). The uncertainties are too high to derive further conclusions from the differences in absolute amounts of these health impacts due to methodological differences (see the supplementary information (SI) for details).

#### Emissions and impacts by sector

#### Electricity from coal as a main driver of impacts

The dominating contributor to greenhouse gas emissions and human health impacts globally is coal power generation and, in particular, *large-scale* coal power generation. Most of the coal power CO$_\text {2}$ emissions are caused in China, where massive amounts of energy are needed for domestic consumption and the production of exported goods. The PM$_\text {2.5}$ and PM$_\text {2.5}$ precursor emissions of coal power plants in China have decreased substantially in recent years due to the introduction of strict emission limits for coal power plants in 2012, but the health impacts still remain high due to the population density and unfavorable environmental conditions like seasonally low wind speeds in combination with cold temperatures. The two countries in the world that stand out even more due to PM$_\text {2.5}$ health impacts are India and Indonesia, which are also densely populated, but additionally feature lenient environmental regulation and struggle with implementing even the existing environmental laws. As a consequence, the flue gas treatment from a major share of coal power plants is far behind international standards. In other parts of the world, such as Western Europe or North America, the general level and enforcement of environmental regulation is more stringent. However, the high standards for new power plants as well as the costs for better quality coal have created a market situation where the operation of new, highly efficient hard coal power plants with lower health impacts is sometimes less profitable than that of old brown coal power plants with much higher emissions. As a result, some of the newest power plants are in the process of being shut down while older, more polluting ones continue to operate^6^. In addition, coal power plants are also often located in or close to population centers under environmental conditions that favor the formation of secondary PM$_\text {2.5}$, so the health impacts from coal power are also high in Europe and North America^7^.

#### Electricity from other fossil fuels

Other types of power plants like gas turbines additionally worsen the situation where they are of old age, located within city boundaries and have low stack heights. Under such conditions, even small oil or gas generating units are responsible for a disproportionately large share of PM-related human health impacts while CO$_\text {2}$ impacts are lower than from coal power plants. Sometimes, inappropriate equipment or operation further contributes to the health impacts, for example in Africa where some emergency diesel generators with low electric efficiencies and no measures for emission reduction are used for baseload power generation. Overall though, modern gas power generation in particular causes significantly lower health impacts than coal power generation in the same location since only NO$_\text {x}$ emissions per amount of electricity generated may be of a similar order of magnitude. Gas power plant impacts come mostly from the US (where natural gas from fracking has replaced the coal as cheapest fossil fuel), the EU (where several countries use natural gas to partly replace coal power generation despite the costs) and Japan (where the role of gas power has increased in the aftermath of the nuclear power plant shutdown from 2011). The global impacts of oil power generation, on the other hand, are difficult to quantify as their use is most common in developing countries with incomplete records of power generation. Oil power is used in these regions because the infrastructure requirements for oil power are relatively low, and in Middle Eastern countries with abundant oil reserves and cheap oil prices.

#### The benefits of co-generation of power and heat

The co-generation of heat and power is a common technique to increase the overall utilization of fuels, although that may decrease the electric efficiency of power plants. For various energy intensive industries such as the chemical industry, this is key to improving process economics. Steam turbines with small electric capacities (below 100 MW), in particular, often have high capacities for heat or steam production. Ignoring the co-generation of heat and power may lead to massive underestimation of related CO$_\text {2}$ emissions, which is a major problem with IEA data^8^ as it has considerable gaps with regards to heat and power co-generation (e.g. in China). Internal combustion engines have high electric efficiencies even in case of small capacities, but their off-heat is of low quality, with a major share of heat being below 100$^\circ$C, so they are rarely used for co-generation in industrial settings with high temperature demands. Instead, they are commonly used for commercial applications (for example in schools, hospitals, airports, or shopping centers) where low-temperature waste heat can be used for buildings in winter, while they are sometimes coupled with chillers to use waste heat for cooling in summer. The complexity of real-world co-generation setups is difficult to capture in this global study as operational data (e.g. on the use of additional steam turbines or auxiliary boilers) or basic parameters (e.g. in the case of heat with several pressure and temperature levels) are generally not available. However, this is unlikely to have a major influence on the global emission patterns in our study as this mostly influences the ratio and quality of heat and electricity outputs of individual units, while playing a comparatively limited role in terms of emissions and human health impacts.

#### Steel-making includes a broad range of polluting activities

Primary steel production has been increasing in developing countries such as China and India in recent years, while primary steel production in the industrialized regions of Europe (like the German *Ruhrgebiet* area) and North America (like in the US *rust belt*) are stagnating or declining alongside increasing imports of steel products. Thus, there is currently an ongoing shift of burden from industrialized to developing countries^9^, but due to a higher susceptibility to PM health impacts in Asia due to high population densities and, to some degree, lower emission standards, the health impacts from primary steel production are increasing. A major share of these health impacts is coming from China (Fig. 2e), where steel mill over-capacities in the past decade have lowered global steel prices and led to higher steel consumption, which, in turn, increases the related impacts even further.

Traditionally, primary steel production has been very labor-intensive so major cities are located close to the largest steel mills. Nowadays, these cities have increased in size even though the number of workers per tonne of primary steel production has decreased. As a result, there are many large steel mills in the immediate vicinity of large numbers of people, which results in very high health impacts per steel mill, even in regions such as Europe, North America or South America (Fig. 2e). The major sources of the regional or local health impacts are the emissions from coking plants and sinter plants, which play central roles with respect to on-site emissions. Basic abatement of primary PM$_\text {2.5}$ emissions from these facilities is common, but more advanced abatement for SO$_\text {2}$ and NO$_\text {x}$ are costly and more difficult to implement. Where the information on flue gas treatment technologies for the sites was incomplete, we assumed average conditions of abatement according to calculation guidelines^10,11^.

Some PM$_\text {2.5}$ emissions are caused by the handling of materials in the coking and sintering processes and thus depend on operational practices as well as proper equipment maintenance, for which there is generally no suitable data available. Hence, real world emission impacts from these types of facilities may deviate and can even be more extreme than those calculated in the present study. While coke is only sometimes prepared off-site, this is much more common for pelletizing. Therefore, we may miss a substantial share of pelletizing impacts from upstream supply chains. Lime production only takes place at some major steel mills, but despite its frequently low stack heights of around 30 m, it plays no major role with respect to steel mill health impacts. The health impacts due to CO$_\text {2}$ emissions from primary steel production mostly come from blast furnaces, which represent a major source of global CO$_\text {2}$ emissions, and to a lesser degree from the converters.

#### Isolated local peaks of oil refining health impacts

Oil refineries are regionally relatively spread out in developed and developing countries, except for few major refining areas such as in Texas or California. The least developed countries do not operate their own oil refineries and rely on imports of oil products, which are often of low quality (e.g. with high sulfur content) and cause higher emissions in power generation, the transport sector and industrial heat supply. The main sources for CO$_\text {2}$ and PM-related emissions from global refineries are the energy supply system of the refinery, the fluid catalytic cracker (FCC) and sulfur production (where they exist). The emissions from the heat supply is thus mostly shaped by the types of fuels that are used rather than the refinery-specific fuel consumption per amount of processed crude oil as the emission intensities of the typical fuels may differ widely. In industrialized countries, the use of natural gas for heat supply is increasing^8^, since the low-value bottom products from refining (such as heavy fuel oil) or petcoke that have been used frequently in the past no longer meet emission standards and would require the installation of costly flue gas treatment systems. Thus, there is a trend to invest in more costly and energy-intensive upgrading technologies, or to sell the petcoke to countries with lower emission standards. For example, a major share of low quality petcoke from US refineries is sold to India, where this type of waste product is used as cheap fuel for cement plants with limited post-combustion abatement.

The FCC and other processes that require catalyst regeneration by combustion are typically the largest single sources of emissions and health impacts in a refineries unless there is proper flue gas control for these processes. Nowadays, many of these have been equipped with electro-dynamic venturi scrubbers (EDVs), which reduce both PM$_\text {2.5}$ and SO$_\text {2}$ emissions and can be further extended to also reduce NO$_\text {x}$ emissions. While the PM$_\text {2.5}$ emission levels of EDVs can be higher than for electrostatic precipitators (ESPs), the other very common PM$_\text {2.5}$ removal technology for FCCs, they have several advantages, including minimal area requirements and limited investment costs. This technology has become very common in the US and China. In most developing countries, the abatement of FCC emissions may be less advanced than in industrialized countries, but we lack respective data.

Another major source of SO$_\text {2}$ emissions can be the sulfur production process (most often a two-stage *Claus* process) when there is no adequate tail gas treatment applied. We found tail gas treatment at most of the recent installations, so there has been considerable progress in eliminating major SO$_\text {2}$ emissions from this process. Emission contributions from flares may also be significant in individual cases (for example when there are longer-term operational problems with the refinery, in the case of poor equipment maintenance, or due to larger accidents). The quantification of health impacts from flaring is challenging since emissions are typically not measured directly and can only be approximated. Improper flare operation may, for example, change the emissions of PM$_\text {2.5}$ by several orders of magnitude^12^ and cannot fully be captured in the present study. Coking and catalytic reforming play no major role for emissions and impacts in our analysis. As refineries are generally very difficult to operate and the equipment is highly specialized, there are only a few companies in the market and most of the modern large-scale refineries follow comparably high standards. Nevertheless, due to their large size, they often represent major sources of health impacts in a region.

#### Impacts of cement production connected to infrastructure build-up

Cement plants are found in most parts of the world and they are also often located close to major cities where cement is needed for construction projects. Thus, their emissions frequently lead to high pollutant exposure for the local population and health impacts can be high, especially in the case of low stacks in combination with large primary PM$_\text {2.5}$ emissions. The highest share of cement plant impacts is observed with regard to modern cements plants in China due to the cement requirements for infrastructure build-up in recent years^9^. Chinese cement plants are major local contributors to impacts, even though their emissions have been reduced by the deployment of state-of-the-art ESPs or baghouses, because of their number and size. The use of high-efficiency kilns with preheaters and precalciners has contributed to emission reductions of CO$_\text {2}$ and NO$_\text {x}$ per amount of clinker produced, not only in China but also in most other countries of the world. Old rotary kilns have mostly been retrofitted with preheaters and precalciners to improve efficiency and plant capacities. We have found that few regions like Russia or Italy still have older types of rotary kilns without preheaters and precalciners in operation. Future reductions of CO$_\text {2}$ emissions are limited by the stoichiometric release of CO$_\text {2}$ from CaCO$_\text {3}$ calcination, which accounts for about two thirds of the current CO$_\text {2}$ releases for modern clinker kilns. This share can only be reduced by carbon capturing or by reducing the share of clinker in cement.

The most common fuels in use, mainly dominated by low quality coal or petcoke, show limited differences in CO$_\text {2}$ emission intensities. Unabated NO$_\text {x}$ emissions are found to be high irrespective of cement plant type even though modern ones typically have somewhat lower NO$_\text {x}$ emissions per clinker output. Thus, post-combustion NO$_\text {x}$ abatement by means of selective non-catalytic reduction (SNCR) has been deployed in most cement kilns of the US, Europe, China and Japan. The emission reduction efficiency of this technology varies based on the amount of NH$_\text {3}$ that is injected. NH$_\text {3}$ is only partly used up, with the remainder being emitted as *ammonia slip*. NH$_\text {3}$ is also a precursor for PM$_\text {2.5}$, so certain trade-offs from SNCR deployment can be observed in the countries where it is used (cf. Fig. 2k). Additionally, high NH$_\text {3}$ concentrations in the flue gas can cause unwanted operational problems, such as the formation of detached plumes. Deployment of more effective NO$_\text {x}$ control by means of selective catalytic reduction (SCR), which simulataneously results in lower levels of ammonia slip and NO$_\text {x}$ in comparison to SNCR, is currently being implemented at about one dozen cement plants worldwide and is expected to increase further. SO$_\text {2}$ emissions are only abated with scrubbers at a few cement plants that would otherwise emit high levels of unabated SO$_\text {2}$, and is generally of lower importance compared to the emissions of NO$_\text {x}$ because limestone inputs to the kiln typically bind some of the sulfur inputs as gypsum.

Due to the unavailability of global kiln input compositions (fuels and raw materials), our model does not capture the full variability of SO$_\text {2}$ emission levels where emissions are not measured and reported. Likewise, the ammonia slip depends very much on the kiln inputs and operational conditions, so they may differ more than we calculate. On-site cement grinding mills are only minor contributors to direct PM$_\text {2.5}$ emissions^11^ and were therefore omitted. Overall CO$_\text {2}$ emissions from our unit-level inventory compare well with a recent world region-level study^13^, but emissions of other pollutants and their health impacts are far different because we include the site-specific types of flue gas treatment, the reported emission measurements, stack heights and the site-specific non-linear calculation of pollutant fate and effects.

#### Health impact reduction potentials

**Figure 2:**
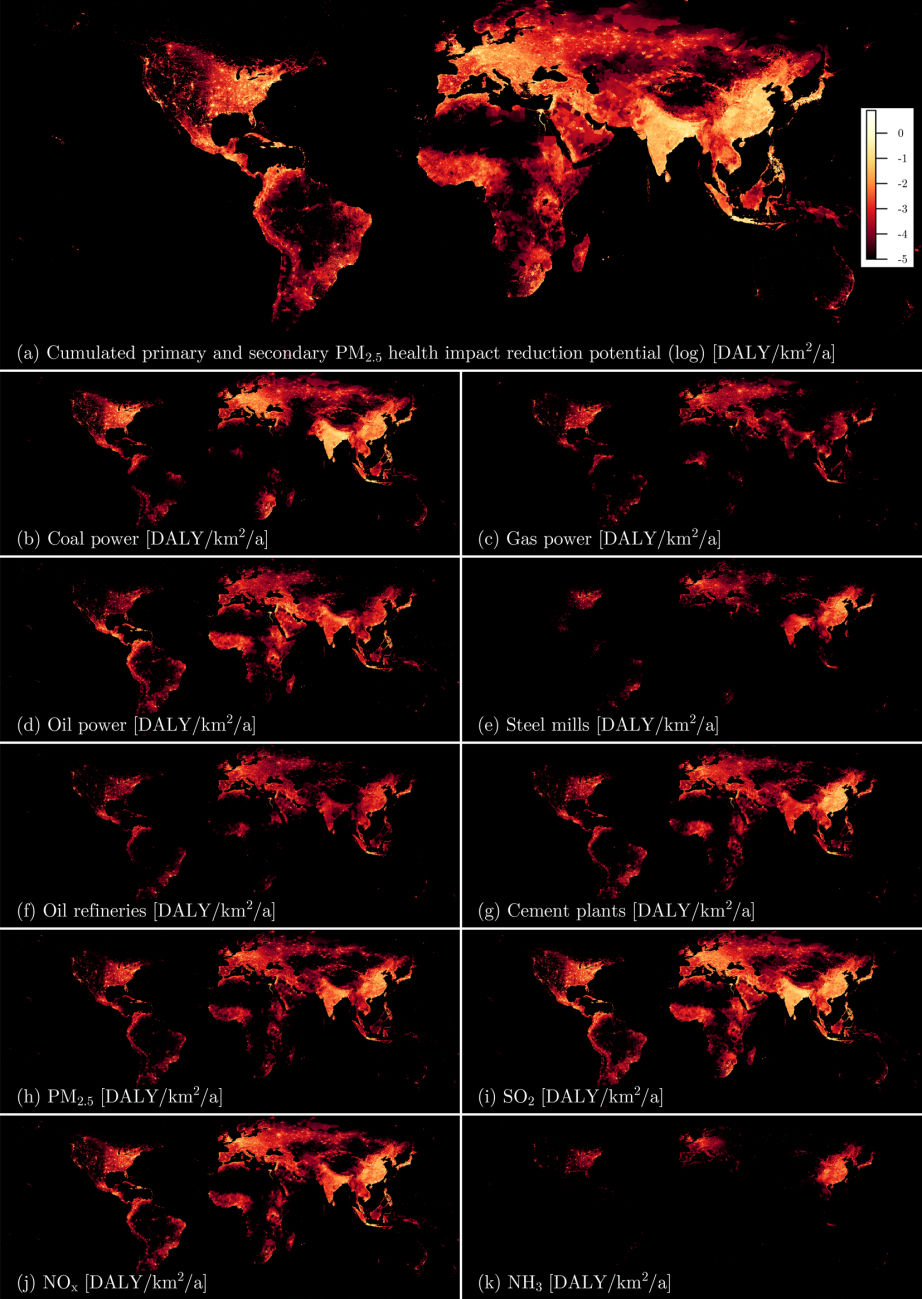
PM health impact maps showing where reductions could occur in the case of full emission abatement (logarithmic scale, annual average CFs, marginal EFs) as (**a**) totals, (**b**-**g**) per emission source and (**h**–**k**) per primary pollutant. Impacts are expressed in [DALY/km$^{2}$/a].

#### Electricity from coal

The main priorities for health impact improvements in the power sector are the abatement of high emissions of CO$_\text {2}$ and SO$_\text {2}$ from coal power generation. While the impacts from both are very difficult to compare due to different time scales, measurability, impact pathways, and non-linearity of human exposure-response to pollutants^14,15^, there is nevertheless ample evidence that both present serious threats to human well-being^1–3,15,16^. The potential benefits from the reduction of coal power SO$_\text {2}$ emissions are highest in North-eastern India and on Java in Indonesia (Fig. 2b, i), where the current coal power plants typically lack any type of post-combustion SO$_\text {2}$ flue gas treatment. Other hotspots of coal power pollution with major impacts are located in South Africa, Mexico and the Philippines, where coal power plants often also feature extreme emission rates of SO$_\text {2}$. In China, Germany, Japan, and parts of the US, there is stricter enforcement of SO$_\text {2}$ emission levels from coal power, but high local or regional population densities still lead to major impacts from coal power SO$_\text {2}$ emissions. As there is usually already contemporary SO$_\text {2}$ reduction equipment installed at the plants in these countries, there remain only the options of switching to fuels with lower sulfur content (e.g. by using higher quality or washed coal), using better sorbents in the flue gas treatment (e.g. by replacing limestone with lime) or by reducing coal power generation in vulnerable areas. High NO$_\text {x}$ emissions from coal power are also of concern, especially from older boilers, in which combustion conditions favor the formation of NO$_\text {x}$.

Limited improvement potentials may be realized by simple changes in combustion conditions, but the achievement of state-of-the-art NO$_\text {x}$ emission levels can typically only be achieved with new low NO$_\text {x}$ burners (LNBs) and post-combustion abatement by SCR. In our assessment, the health benefits from NO$_\text {x}$ emission reduction outweigh the observed NH$_\text {3}$ slip from SCR operation. Primary PM$_\text {2.5}$ health impacts from large-scale coal power generation are nowadays of less concern than SO$_\text {2}$ and NO$_\text {x}$ emissions due to effective post-combustion emission reductions and high stack heights. Nevertheless, a study^17^ indicates that there may be a large amount of outdated PM reduction equipment still in operation in places such as India (97.9% in 2010) or Russia (57.0% in 2010). In fact, satellite imagery reveals that the ESPs of many coal power plants in these countries are long and have small cross-sectional areas. Modern ESPs, however, have much larger cross-sectional areas and are shorter to reduce the flue gas velocity, aid in the deposition of particles, and prevent their re-entrainment^18,19^. While our study lacks detailed measurement data for this as evidence, there is still the chance that such units emit disproportionately high amounts of primary PM$_\text {2.5}$ and would thus present a highly attractive option for further health impact reductions at limited costs.

Several industrialized countries are currently pursuing long-term strategies to quit coal power generation in order to reduce CO$_\text {2}$ emissions and associated risks for human health, but the short and mid-term consequences of these strategies may reduce investments in existing plants and sometimes even lead to the premature closures of the newest, most efficient units (like the *Westfalen* or *Moorburg* plants in Germany^6^), while older units with less effective flue gas treatment and lower quality fuel inputs (such as most German lignite power plants) are cheaper to run and thus remain in operation. Moreover, if such coal power exit strategies are accompanied by a relocation of energy-intensive industries to countries with over-capacities of cheap coal power (like in the case of the steel or chemical industry to China in recent decades), such strategies might be rendered globally ineffective. Thus, we suggest assessing fossil power exit strategies from a human health perspective with a site-specific time-series comparison beyond country boundaries.

#### Electricity from natural gas

The impacts of well-maintained gas power plants per amount of electricity generated are typically substantially lower than for coal power both due to lower relative CO$_\text {2}$ emissions and PM-related emissions. Additionally, the units are typically smaller in capacity and more widely distributed so that improvements at the individual unit level may bring fewer benefits in terms of human health impacts. The most beneficial upgrades can come from very old units with high NO$_\text {x}$ emissions located in cities. Such units are still in operation in developing countries like India and in major oil-producing countries in the Middle East, where there is an abundant supply of cheap natural gas as a co-product of oil production (Fig. 2c). Water or steam injection into the combustion chambers of such units may already reduce peak temperatures and thus NO$_\text {x}$ formation significantly (in the range of up to 90%^20^), but higher reductions are achievable with post-combustion measures like SCR. Most major gas turbine manufacturers also offer upgrades for their gas turbines that can help to restore degraded electrical efficiencies of old units and may even allow such units to achieve higher efficiencies than when they were new. Such upgrades may become economically and ecologically attractive since the relative fuel consumption and CO$_\text {2}$ emissions decrease. A challenge for NO$_\text {x}$ emission reductions from gas power generation are units used for secondary frequency control since sudden increases in electricity generation of such gas turbines may be accompanied by spikes in NO$_\text {x}$ emissions until the abatement technologies reach their design conditions. We observe that such gas generating units are typically of smaller capacity and their utilization over the year is lower than for baseload gas power generation, so this still results in comparably low health impacts. Another special application of gas turbines is the co-generation of electricity and desalinated sea water in Middle Eastern countries. Abundant solar irradiation and the operational flexibility of existing gas power generation would generally make the combination of photovoltaics with gas power an attractive option to reduce environmental impacts in such regions. This would require a complete shift to reverse osmosis plants for sea-water desalination, however, since the off-heat from existing gas turbines is today an essential input to seawater desalination.

#### Electricity from oil

The low costs of photovoltaic modules are an option for developing and least developed countries to substitute some of their polluting decentral oil power generation we observe (cf. Fig. 2d), which is largely related to the sulfur levels in the fuel. These sulfur levels originate from refiners that sell their lowest quality fuels to the least developed countries without their own refineries. Major progress on sulfur levels has been made for transportation fuels in recent years (mostly in terms of car diesel^21^ and fuel oil for ships^22^), but it is unclear to what degree progress might also have been made with respect to industrially used diesel, light fuel oils and heavy fuel oils is unclear, as there has been no systematic global assessment of this to date. Individual accounts, however, indicate that industrially used fuels still commonly contain substantial amounts (in lower single-digit percentages), which has also been assumed in the emission estimates of this study. As post-combustion abatement due to the small size of most oil-fired units is uneconomical and technologically difficult, there remains only fuel desulphurization as a realistic option for SO$_\text {2}$ emission reductions. This is already practiced for a broad range of refinery products around the world and the removed sulfur can often even be turned into a salable product, but this may require governmental intervention to realize. As we observe that SO$_\text {2}$ impacts reach far beyond their emission source, there would typically also be some incentive for the regulators in the oil-refining countries to prevent high sulfur contents in any refinery product. Large-scale oil-burning steam turbines have become rare due to unfavorable economic conditions, and can now mostly be found in Japan. The emission abatement options for such units are generally the same as for large-scale coal generating units and have, in many cases, already been implemented in Japan, but the main problem in Japan is often the very high population density as emission levels are already low. Under such circumstances, the technical options for health impact reductions have largely been exploited and a switch of technology would be required for further improvements. Further quantitative country-level analyses are provided in the SI.

#### Primary steel-making

The steel mill health impacts from greenhouse gas emissions are largely due to the use of coke and coal as reduction agents in the blast furnace. Technically, hydrogen (H$_\text {2}$) gas has been identified as a viable substitution option^23^, but due to high prices this strategy is currently incompatible with the demand for cheap primary steel in the construction and transport sectors. In particular, current H$_\text {2}$ production is fossil fuel-based as water electrolysis with renewable energy and other renewable process options are still too costly. Even with continuous access to very cheap renewable electricity, the electrolysis cells scale linearly with the required capacity so there can only be very limited economic and ecologic benefits due to scaling effects. At the same time, this technology competes with a combination of fossil-derived H$_\text {2}$ and carbon capture and sequestration (CCS), which might be economically more attractive. A higher share of steel recycling might also help to reduce the more energy-intensive primary production, but it cannot meet the currently increasing demand for steel^24^ and may in future reach limits at a point where alloying materials become technologically difficult to separate from the steel^25^. A partial substitution of steel as a construction material might be a long-term option, but PM$_\text {2.5}$, NO$_\text {x}$ and SO$_\text {2}$-related human health impacts can be reduced more directly where these emissions and their impacts are based on fuel qualities and handling, so the regular techniques for combustion control (primary measures such as control of combustion temperatures) and post-combustion control previously mentioned (scrubbers, filters or conversion of pollutants) apply. Such measures generally allow emissions to be reduced by 90% or more and would yield major benefits in China and India (Fig. 2e). Management practices of coke ovens and sinter plants may also cause major emissions from diffuse sources that are more challenging to control. In particular, primary PM$_\text {2.5}$ emissions from diffuse sources may affect local communities due to their low emission heights, but several examples in Japan, China, and Europe exist that demonstrate substantial improvements for controlling diffuse emissions^26^. A critical problem will remain the locations of individual steel mills within cities, where these cities have grown around them. The relocation of entire steel mills, as practiced in a few cases in China, is in most cases unrealistic and unattractive for the cities as they would lose tax income. Our study indicates that phasing out outdated equipment and reducing over-capacities present major options for improvement in China and India as well as in many other countries, while detailed environmental impact assessments coupled with careful zoning prior to granting permission for new installations may likewise be tools to steer progress. An essential prerequisite for such targeted measures is the implementation of regular exposure concentration measurements.

#### Oil refining

Due to major improvements in various processing steps, we find that the energy supply of refineries now presents one of the major levers for emission and health impact reduction. The actions of the *U.S. Environmental Protection Agency* (EPA) in the implementation of *New Source Performance Standards* (NSPS) for industrial boilers and oil refineries constitute one example on how to assess country-wide emission sources from oil refineries and to continuously reduce them. Thus, the formerly central role of FCCs for pollutant emissions from oil refineries in the US could be reduced substantially due to the introduction of particulate control, SO$_\text {2}$ scrubbing and combustion measures that reduce NO$_\text {x}$ emissions. Likewise, most global FCCs at this point have relatively stringent control of FCC emissions, with additional potential mostly coming from the installation of SO$_\text {2}$ scrubbers (in case high sulfur contents in the combusted materials cause high SO$_\text {2}$ emissions) as well as from the implementation of NO$_\text {x}$ emission reduction by SCRs. Most impacts concerning human health are calculated for the refineries in the vicinity of Los Angeles, San Francisco, Guangzhou, Tokyo, Osaka and Jakarta (Fig. 2f) as the population in these places is extremely vulnerable to PM and the local refineries are located very close to or within those cities. The refineries around Jakarta, which are more than 300 km away from the city, represent an a exception since their impacts are largely caused by their high pollutant emissions instead of their proximity to cities. The main emission sources in these refineries include flaring, which may be an indicator of larger operational problems. A challenge in the implementation of post-combustion abatement for refineries will be the number of emission sources and how they are emitted. The Japanese regulation, for example, frequently asks for high stacks (heights above 100 m), which not only helps to keep ground-level pollutant concentration peaks low, but also combines several pollutant sources into a small number of large stacks. Post-combustion abatement measures can be implemented mory economically under such circumstances in comparison to refineries with large amounts of distributed emission sources and individual small stacks (heights below 50 m), which are for example common in the US. Sulfur recovery in the case of refineries with high-sulfur crude feedstock can also be the major source for SO$_\text {2}$ pollution when there is no effective tail gas treatment of Claus process off-gases. All recently installed sulfur recovery units for which we have found public records seem to include some kind of tail gas treatment unit. Still, it is likely that not all smaller and older units have it installed, but we lack data on that. Where such treatment is missing, its introduction should be a priority, especially as there is a broad range of technologies with total sulfur recovery rates exceeding 99.9% available for that purpose^27^. In terms of the health pollution impacts from the refinery heat supply, the solutions are not quite as straightforward. According to International Energy Agency (IEA) data, most refineries already use refinery gas, a mixture of volatile by-products of oil refining, and natural gas as main refinery fuels^8^. These fuels are both comparably low in SO$_\text {2}$, PM$_\text {2.5}$ and CO$_\text {2}$ emission intensity. A lower demand for highly polluting refinery bottom products such as heavy fuel oil will in future increase relative refinery fuel demands per amount of crude oil input. Certain impact improvements are still possible by more frequent deployment of LNBs, possibly in combination with SNCRs or SCRs. Concepts for replacing high-temperature heat as input in oil refining with electricity derived from solar or wind power are also under development, but depend on new catalysts and require major process changes. Thus, the most effective health impact reduction pathways for refinery heat supply will require further research, that also takes changing future demand patterns for oil products into consideration.

#### Cement-making

Major issues of global cement plants in terms of their human health impacts are the CO$_\text {2}$ emissions caused stoichiometrically by the calcination of CaCO$_\text {3}$ (about 60% of total CO$_\text {2}$ emissions) and, to a smaller degree, by the carbon content in the fuel input. An overall reduction of both is to a certain degree possible by reducing the clinker share in cement, but the former global trend in this direction has been plateauing^28^. A switch to natural gas or other fuels with lower CO$_\text {2}$ emission intensity might bring some additional saving potentials at increased costs, but this also only addresses a minor part of the problem. More savings might be possible by substituting the coal and petcoke inputs of the cement kilns with wastes that would otherwise be burned in waste incineration plants or land-filled. Two central requirements for this, however, would be that the clinker kilns have sufficient air pollution control to deal with all the pollutants that are emitted (like heavy metals, dioxins, etc.), and that the cement product is also not being contaminated with pollutants (which could otherwise cause human health impacts via different impact pathways). The calcination CO$_\text {2}$ releases, however, can only be reduced through decreased clinker demands or post-combustion CO$_\text {2}$ capturing. As government investments in the construction sector are commonly used to prevent slowdown of local economies, and many developing countries will have major infrastructure requirements in the coming years, a reduced clinker demand seems unlikely and post-combustion CO$_\text {2}$ capture remains the only realistic mid-term option. The other major driver of cement plant human health impacts are the high NO$_\text {x}$ emissions, even in the case of commonly used SNCR abatement. Here, the global cement industry is lagging behind in technological development as the use of SNCR with average removal rates of 40% is not common anymore in the power sector due to its limited effectiveness and the high NH$_\text {3}$ slip, which not only contributes to PM human health impacts via the formation of NH$_4$NO$_\text {3}$ and (NH$_4$)$_{1.5}$SO$_4$, but may also cause severe operational problems. The implementation of the superior SCR (with NO$_\text {x}$ removal rates up to 95%) is underway for a small number of cement plants, and the installation should be sped up in regions with major NO$_\text {x}$ impacts from cement production (especially in China, Europe and Japan, as visible in Fig. 2g). Plants in the rest of the world often do not even have SNCR installed and may directly go for SCR, but the benefits from SNCR in these regions usually outweigh the additional damage from NH$_\text {3}$ slip and might thus present an acceptable short-term solution at lower costs. Finally, it is very common among cement plants to use clinker coolers with moving grates, which are frequently attached to separate stacks with low heights and small ESPs with few stages and low flue gas residence times. The primary PM$_\text {2.5}$ emissions from these coolers are a major contributor to cement plant human health impacts where cement kilns are situated close to major cities as the emissions from these coolers can be high and may cause local PM$_\text {2.5}$ concentration peaks due to their low stacks (Fig. 2h). Most modern cement plants, for example in Europe, Japan and sometimes in China, use all of the hot air from the coolers for preheating, which reduces the cement plant coal or petcoke fuel needs, decreases CO$_\text {2}$ emissions, improves plant economics and helps with the air pollution. As is the case of power generation or steel-making, reducing the consumption of coal and similar fuels is the key to lowering human health impacts.

### Materials and methods

#### General concept

The emission inventory in this study uses reported emission data where possible. Overlapping data sources are ranked by their data quality for a given region and are prioritized accordingly. The formats of the various data sources are aligned and a broad range of emission models are used as a last resort to fill emission data gaps and to disaggregate different emissions per emitting site. These models use engineering calculations based on mass and energy balances where feasible to preserve links between emissions and the technical parameters of equipment and inputs rather than being based on purely empirical approaches (such as in^29,30^). The use of these engineering models is possible due to comprehensive primary data collection on the technical specifications of each site as well as the linking of numerous databases with technical data.

The system boundaries enclose each emitting site with the different types of facilities (power plants, steel mills, oil refineries and cement plants) separated from each other, even if they are physically located within the same battery limits. Downstream and upstream emissions and impacts are not included to avoid double-counting (please see the SI for more details on the possible consequences). In addition, each site was sub-divided into smaller units (like generating units of power plants or major process sections in the case of oil refineries) where that is possible. The emitted substances covered are CO$_\text {2}$, PM$_\text {2.5}$, SO$_\text {2}$, NO$_\text {x}$ and NH$_\text {3}$. The temporal scope of this study is the year 2016, with data having partly been extrapolated from 2015 or 2017 due to data gaps. In a few countries (India, Australia, South Africa), emissions or activity data was reported for fiscal years instead of calendar years. In these cases, calculations are performed for the fiscal year 2016/2017. Site-specific activity data is used if it is reported, or else it is estimated from other plant-level data or through a combination of national statistics and capacities (in that order of priorities).

#### Emission sites

The backbone of this study is an emission site database with basic technical data for each site, to which data from a large number of other databases is added. The power plant part of this emission site database comes from the commercially available Platts World Electric Power Plant (WEPP) database^31^, which provides data on 125,000 coal, gas and oil power generating units and is globally comprehensive except for very small units (like emergency diesel generators) and for China, where the database provider has difficulties keeping up with the fast rate of power plant construction and the amount of small, unregistered units. For steel-making, the emission site database covers all the steel mills with blast furnaces from a number of reports on global steel-making capacities^32,33^ and was supplemented by industryabout data^34^ and (especially in the case of China) by a large number of individual entries from company and governmental homepages. The basic oil refinery data was obtained from literature^35^ for 2016 and finally, the cement plant data was obtained by combining grey clinker plants with rotary kilns from industryabout^34^, the Global Cement Directory 2017^36^ and further manual additions.

For each of the emission sites, coordinate data has been collected. Manually verified coordinates are used for large power plants, all steel mills, all oil refineries and the largest cement plants. Other site coordinates are filled from a large number of databases where possible^34,37–47^. The remaining units without coordinates obtained their coordinate data automatically^48^ based on unit and location names (only necessary for small power plants globally and small Chinese cement plants).

#### Reported emission data

Reported emission data was obtained from a large number of national and super-national data sources. Among them were pollutant release registers such as E-PRTR for the European Union^37^, NPI and NGER for Australia^49,50^, NPRI and GHGRP for Canada^47,51^, and NEI and GHGRP for the US^52,53^. Databases from the China Electricity Council^54–56^ are used to derive Chinese power plant emissions through a combination of activity data and reported flue gas concentrations with a calculation procedure from an earlier study^39^. Data by the Indian Central Electricity Authority provides CO$_\text {2}$ emissions in combination with activity data for Indian power plants^57^. US emission data^52,53^ is furthermore supplemented with eGRID data^46^ and the recent eGRID add-on for PM$_\text {2.5}$^58^. For South Africa, Eskom activity data is available for the fiscal year 2016/2017^59^ and can be combined with 2017/2018 flue gas pollutant concentration data^60^. The linking of emission data sources and basic site data^39^ is semi-automatically extended by correlating basic unit data and checked manually. Validation for modeled data in contrast to reported data is shown in the SI.

#### Power plant approach

In the case of the baseline database for power generating units, a large number of about 8000 manual corrections and updates have been applied to individual data entries. The technical data therein is supplemented by several databases^38,39,41–45,54–57,59,61–68^ that provide further information on plant setups, operating parameters, technologies in use and, in particular, the co-generation of power and heat. Literature data^31,38^ as well as the company homepages of major generating unit suppliers (General Electric, Siemens, Mitsubishi Hitachi Power Systems, Harbin, etc.) are used to derive default gap-filling rules based on recent developments in terms of steam reheating, regeneration and cooling systems, as described in the SI.

Detailed modeling of steam turbine types includes specific steam cycles, regeneration, co-generation of heat and power, backpressure and condensing turbines, steam temperatures, steam pressures, cooling temperatures and operation parameters (part-load efficiencies, boiler behavior, heat-to-power (HTP) ratios) and thus represents a substantial expansion of an earlier approach^68^. Thermodynamic data for the calculation of gas turbine and internal combustion engine efficiencies is generally not available from any of the above-mentioned data sources, so instead a database of gas turbine and internal combustion engine efficiencies at ISO conditions for electricity grid frequencies of 50 or 60 Hz is built up from 1414 technical data sheets and other performance records, as listed in supplementary table 26. Where the type of gas turbine or internal combustion engine is known, the thermodynamic efficiency can be used directly, while for the other units, type-specific and size-specific correlations are derived as fallback (see the SI for details). Environmental data (temperatures and humidity^69^) and operational data is then combined with the derating curves^70–73^ to adapt the efficiencies for site-specific local conditions.

Flue gas treatment data for each unit is used where reported^31,39,54–56,61^ and from manual additions with satellite imagery as described before^39^), while the remaining gaps are filled with default assumptions adapted an earlier study^39^ and power plant database documentation^31^. Site-specific HTP ratios are used wherever possible^41,46,65–67^ in combination with company homepages. For gas turbines, internal combustion engines and combined cycle power plants, HTP ratios from the data source mentioned above typically fall within a relatively narrow range independent of unit size, so any gaps in the case of these units can be filled with technology-specific averages, which we derived from that data (0.69 for combined cycle power plants, 1.73 for gas turbines, and 1.10 for internal combustion engines). HTP ratios of steam turbines vary within a large range (standard deviation 3.3) as the main purpose of some is electricity generation while others mainly supply heat. We observe that this somewhat depends on the electric capacity of each unit. Units with small electric capacities tend to have high HTP ratios and large units tend to have small HTP ratios. Thus, we derive a unit size based correlation from a fit to reported HTP ratio data and apply it to the cases where HTP ratios are unknown (as shown in the SI). To ensure feasibility of the results, HTP ratios are cross-checked with the typical ranges per technology provided by^74^ and are found reasonable. The use of national HTP data per fuel from^8^ has also been investigated but the quality of data was too low to be used (partly technically infeasible data).

Coal properties per unit are generally obtained from^39^, while average ash contents in China and India are scaled to more recent 2016 conditions (see the SI for details). Where unit-level data was not available, the default fuel-specific ash and sulfur assumptions from^10,11,75^ are used, while lower heating values and CO$_\text {2}$ emission factors are obtained from^76^. Averages are calculated where two main fuels are reported. The sulfur content of oil types is derived from 2016 data in the case of diesel^21^, while heavy fuel oil and light fuel oil sulfur contents are estimated with the sulfur contents listed in supplementary table S2.

Net power generation data is added from various databases^46,54–57,59,63^. In the case of data gaps, it was calculated from reported annual CO$_\text {2}$ emissions, emission factors and electric efficiencies (as described before^39^), or else approximated from national statistics per fuel type and corresponding total operational generating capacities^8^. Fuel inputs are derived from net power generation data and efficiencies. Unabated power plant emissions for PM$_\text {2.5}$, SO$_\text {2}$, NO$_\text {x}$ and NH$_\text {3}$ are then calculated based on Tier 2 emission factors per fuel input^10^ by reverting the default abatement assumptions used there and by scaling SO$_\text {2}$ emissions proportionally to plant-specific and average fuel sulfur contents. The emission factor for gas turbine NO$_\text {x}$ emissions is used from reference^75^ because the value in reference^10^ does not match the company-reported ranges^20^. The unabated emissions are sequentially abated with non-linear flue gas treatment models for each flue gas treatment step derived from two data sources^77,78^ as described in the SI. In the absence of ammonia slip emission factors for SNCR and SCR NO$_\text {x}$ abatement, values of 10 ppmvd and 2 ppmvd at 15% O$_\text {2}$ are assumed, respectively (based on several sources^78–80^). In cases where there is subsequent wet flue gas treatment, we assume that NH$_\text {3}$ emissions are reduced by 84%^81^. Abated combined cycle power plant emissions are then passed from gas turbines or internal combustion engines to their heat recovery steam generators (HRSGs) and the associated steam turbines based on steam turbine electricity generation shares. HRSG flue gas emissions are then reduced further based on the HRSG flue gas treatment. Emissions from auxiliary firing of HRSGs are not added in the model unless specifically reported in any of the emission databases mentioned above. The associations of HRSGs and gas turbines or internal combustion engines are set automatically based on the names of the units. Stack heights for the resulting abated emissions are added from literature^61,82^, our own measurements based on elevation data^83^, and correlations from reported stack heights, as described in supplementary table S2.

#### Steel mill approach

The steel-making covered explicitly by this study is primary steel-making with blast furnaces, which accounts for 74.0% of global steel production in 2016^24^. Secondary steel-making with electric arc furnaces, in contrast, accounts for 25.5% of global steel production^24^, but its environmental impacts are heavily shaped by electricity consumption^84^, so its health impacts are implicitly covered by fossil power generation. Capacities of steel mills are interpolated linearly for 2016 if only capacities for other years are known. Where no capacity is known (mostly for some Chinese mills), the capacity is approximated from the number of blast furnaces of the steel mill and the average blast furnace capacity of 1.31 Mt ${a^{-1}}$, the average of known capacities per blast furnace. Other types of on-site facilities are identified from satellite imagery for each steel mill (coke ovens, sinter plants, pellet plants, lime kilns, basic oxygen furnaces, open hearth furnaces). The default assumption is that sinter plants and basic oxygen furnaces are present unless otherwise visible as this is clearly the most common case. Steel mill utilization is then calculated from national statistics of pig iron production^24^ and specific steel mill capacities. The activity data of the other on-site equipment is approximated from input requirements per amount of pig iron production^10,84^ (details in the SI). Emissions for each piece of equipment are then calculated according to guidelines^10,76,85^ with additions^11^ in the case of data gaps like for PM$_\text {2.5}$ emissions of lime kilns. For coke ovens, we have identified whether they recover by-products with satellite imagery. This data is required to determine the coke oven emission calculation procedure for each site, and by-product recovery was assumed as a default assumption for blurry satellite imagery. Average emissions are used in the case that the emission factors differed per type of fuel (coke oven gas, blast furnace gas) as their shares per site are unknown. Emission control is set to *effective* or *modern* for industrialized countries based on stricter emission limits, while emission control is assumed *moderate* or *conventional* in developing and least developed countries. Site-specific stack heights for blast furnaces, basic oxygen furnaces, open hearth furnaces, coke ovens, sinter plants, pellet plants or lime kilns are used where they are known^82^ or can be measured with external data^83^, and otherwise they are derived from correlations based on the steel mill size and the country (as documented in supplementary table S2).

#### Oil refinery approach

The capacities of refinery equipment at the end of 2016 for each refinery are known^35^ and are supplemented by manual additions and corrections. The average utilization of each piece of refinery equipment is approximated using national data on crude oil processing^8^. Manual data collection based on satellite imagery and company homepages adds site-specific data on the flue gas treatment of fluid catalytic crackers. The data on sulfur production technologies^86^ as well as from company homepages is merged with the refinery database, with the default assumption in the case of data gaps being a two-stage Claus process. Subsequent tail gas treatment is also added from these data sources. The energy balances and national fuel mixes for heat supply^8,87^ are added, which then allow corresponding emissions to be calculated^10,76,85^. Sulfur levels in fuels are adjusted to national averages as in the case of power plants. For refinery equipment with additional emissions beyond fuel consumption (fluid catalytic cracking, coking, reforming, sulfur production, bitumen production, flaring), several guidelines^10–12,27,88^ are used. Site-specific abatement of emissions by means of flue gas treatment was calculated with the data in supplementary table S3^89^. Satellite-based VIIRS flaring amount measurements for 2016^90,91^ are added per refinery. Where refinery-specific flaring amounts are unknown, we use national averages per crude input from known amounts or else averages for developed, developing or least developed countries. Stack heights for fluid catalytic cracking, flares and other types of equipment are site-specific where directly available^82^ or measurable^83^, or else approximated with national and global correlations, as detailed in supplementary table S2.

#### Cement plant approach

The cement plant model covers global gray clinker production from rotary kilns, so the small share of much more expensive white clinker production^92^ and the production of gray clinker in shaft furnaces (about 2% of the total gray clinker amount^28^) are neglected. In the case that the gray clinker capacity of a cement plant is unknown, it is approximated from its number of rotary kilns and the average rotary kiln capacity of 1.36 Mt $a^{-1}$ (based on the average of reported capacity data). Exact locations of the cement plant locations are identified from the data sources mentioned above and for small cement plants in China (<20% of the Chinese capacity) automatically^48^. Site-specific flue gas treatment data is derived from satellite imagery and company homepages where possible, and NO$_\text {x}$ abatement by SNCR is furthermore set as default assumption for China and Japan (based on several data sources^11,93,94^). As rotary kilns with preheater and precalciner are the dominating type globally^28^, this setup is used as a default assumption unless a different setup is identified from satellite imagery. Kiln utilization is calculated from cement and clinker production data^28^ with gap-filling and disaggregation using national 2016 cement production data^92^. Corresponding fuel inputs per tonne of clinker and fuel ratios (coal/biomass/wastes) are then also added from national statistics^28^. The resulting fuel combustion emissions (including calcination) and clinker cooling emissions (from particle entrainment during cooling) are subsequently calculated according to guidelines^10,76,85^ with adaptations for flue gas treatment types and kiln types^11^, as described in more detail in the SI. The stoichiometric CO$_\text {2}$ emissions are added based on national statistics^28^ and cross-checked against global default values^85^. National cement plant SO$_\text {2}$ emission intensities^84,94^ are used where available, while NO*x* emission intensities per kiln type are used from another data source^11^. Primary PM$_\text {2.5}$ emissions are also used from^11^ because other literature data^10^ does not distinguish clinker cooling and kiln emissions despite separate stacks, different stack heights and types of flue gas treatment technologies in use. Cooler PM$_\text {2.5}$ emissions are set to zero where no cooler stacks are identified on satellite imagery (e.g. in the case of cooling air use for preheating or planetary coolers instead of the more common moving grate coolers). Diffuse primary PM emissions from cement handling and further processing (e.g. screening, crushing, milling) are typically between 1 and 4 orders of magnitude lower than from the kilns and additionally lack PM$_\text {2.5}$ share information in^11^ so they are excluded. Stack heights are site-specific for clinker coolers and kilns where elevation data is available^83^ or else derived from capacity-based national and global correlations, as described in supplementary table S2. Ammonia slip emission factors in the case of SNCR and SCR NO$_\text {x}$ abatement are not available and are thus assumed to be 40 mg Nm$^{-3}$^95^ and 5 mg Nm$^{-3}$^96^, respectively.

#### Atmospheric fate model and health impacts

A literature model^97^ is used to derive site-specific characterization factors (CFs) for primary and secondary particulate matter health impacts. The background data in this model (atmospheric conditions, population) reflects 2015 conditions (in contrast to the 2016 emission data). Deviating from the original approach^97^, the transitioning radius from the high-resolution to the low-resolution model part around each emission source is increased to 1000 km (up from 250 km) as it has been noted that otherwise only a limited part of the total health impacts can be covered for very high stacks at a detailed resolution. The model is fed with emission locations and stack heights as described above. Emission amounts are combined if they occur at the exact same location (longitude, latitude and height). Calculations are performed for 1000 Monte Carlo runs randomly altering the atmospheric conditions, which then allowed health impact maps to be derived based on emission sources and pollutants, as shown in Fig. 2. This approach includes global non-linear dose-response functions for five types of PM-related human health impacts: ischemic heart disease, stroke, lung cancer, chronic obstructive pulmonary disease and lower respiratory infections. The model furthermore includes the atmospheric chemistry that can transform emissions of SO$_\text {2}$, NO$_\text {x}$ and NH$_\text {3}$ into secondary PM$_\text {2.5}$. The health impacts from CO$_\text {2}$ emissions are quantified with the *human health core* characterization factors from^14^ for both fossil and biogenic CO$_\text {2}$ due to common long re-growth times of biomass^98^. The maximal health impact reduction potentials are quantified by assuming full abatement of all emissions.

### Supplementary Information

Supplementary Information 1.

Supplementary Information 2.

## Data Availability

Extended descriptions of methods and additional results are available in the SI. The source code for the model (R v3.6.0)^99^ and data are available from http://dx.doi.org/10.17632/k2vgcm4bnk.1^100^. The site-specific emission and health impact data is fully compatible with the power plant data sets from^31,38,39,68,97^. For additional data requests, please contact the authors directly at cobersch@ethz.ch.

## References

[1] World Health Organization (WHO). Ambient (outdoor) air quality and health (WHO) (2018). http://www.who.int/news-room/fact-sheets/detail/ambient-(outdoor)-air-quality-and-health.

[2] GBD 2019 Diseases and Injuries Collaborators. Global burden of 369 diseases and injuries in 204 countries and territories, 1990–2019: A systematic analysis for the Global Burden of Disease Study 2019. *The Lancet* **396** (10258), 1204–1222 (2020). 10.1016/S0140-6736(20)30925-9 PMC7567026 33069326

[3] Intergovernmental Panel on Climate Change (IPCC). Climate change 2013: Synthesis report. In *Contribution of Working Groups I, II and III to the Fifth Assessment Report of the Intergovernmental Panel on Climate Change IPCC* (2013).

[4] Crippa M, Guizzardi D, Muntean M, Schaaf E, Dentener F (2018). Gridded emissions of air pollutants for the period 1970–2012 within EDGAR v4.3.2. Earth Syst. Sci. Data.

[5] United Nations Framework Convention on Climate Change (UNFCCC). Greenhouse Gas Inventory Data - Detailed data by Party (2020, accessed 6 Dec 2020). http://di.unfccc.int/detailed_data_by_party.

[6] Bundesnetzagentur. Results of first tendering process to reduce the production of electricity from coal (2020, accessed 6 Dec 2020). https://www.bundesnetzagentur.de/SharedDocs/Pressemitteilungen/EN/2020/20201201_Kohle.html.

[7] European Environment Agency (EEA). The European environment—state and outlook 2020 - Knowledge for transition to a sustainable Europe (2020). https://www.eea.europa.eu/ds_resolveuid/301fc55a26a943bc8de1cd5d17d7ec66.

[8] International Energy Agency (IEA). IEA Extended World Energy Balances 2018. Paris (2018, accessed 3 Dec 2018). 10.1787/data-00513-en.

[9] Oberle, B., Bringezu, S., Hatfeld-Dodds, S., Hellweg, S., Schandl, H. *et al*. Global resources oOutlook 2019: Natural resources for the future we want (2019). http://hdl.handle.net/20.500.11822/27517.

[10] European Environment Agency (EEA). EMEP/EEA air pollutant emission inventory guidebook 2016 (2016). https://www.eea.europa.eu/publications/emep-eea-guidebook-2016.

[11] U.S. Environmental Protection Agency (EPA). Air Emissions Factors and Quantification—AP 42, Fifth Edition Compilation of Air Pollutant Emissions Factors, Volume 1—Stationary Point and Area Sources (2011). https://www.epa.gov/air-emissions-factors-and-quantification/ap-42-compilation-air-emissions-factors#5thed.

[12] Australian Government—Department of the Environment. National Pollutant Inventory Guide v6.1 (2015). http://www.npi.gov.au/resource/national-pollutant-inventory-guide.

[13] Miller SA, Moore FC (2020). Climate and health damages from global concrete production. Nat. Clim. Change.

[14] Verones F, Hellweg S, Antón A, Azevedo LB, Chaudhary A, Cosme N, Cucurachi S, de Baan L, Dong Y, Fantke P, Golsteijn L, Hauschild M, Heijungs R, Jolliet O, Juraske R, Larsen H, Laurent A, Mutel CL, Margni M, Nunez M, Owsianiak M, Pfister S, Ponsioen T, Preiss P, Rosenbaum RK, Roy PO, Sala S, Steinmann Z, van Zelm R, van Dingenen R, Vieira M, Huijbregts MAJ (2020). LC-IMPACT: A regionalized life cycle damage assessment method. J. Ind. Ecol..

[15] Burnett R, Pope A, Ezzati M, Olives C, Lim S, Mehta S, Shin H, Singh G, Hubbell B, Brauer M, Anderson H, Smith K, Balmes J, Bruce N, Kan H, Laden F, Pruss-Ustun A, Turner M, Gapstur S, Diver R, Cohen A (2014). An integrated risk function for estimating the global burden of disease attributable to ambient fine particulate matter exposure. Environ. Health Perspect..

[16] World Health Organization (WHO). WHO Air quality guidelines for particulate matter, ozone, nitrogen dioxide and sulfur dioxide—Global update 2005—summary of risk assessment. Geneva (2006). http://apps.who.int/iris/bitstream/handle/10665/69477/WHO_SDE_PHE_OEH_06.02_eng.pdf?sequence=1.

[17] Tong D, Zhang Q, Davis S, Liu F, Zheng B, Geng G, Xue T, Li M, Hong C, Lu Z, Streets D, Guan D, He K (2018). Targeted emission reductions from global super-polluting power plant units. Nat. Sustain..

[18] Babcock & Wilcox. Basics of Electrostatic Precipitator (ESP) Operation (2020, accessed 9 Dec 2020). https://www.babcock.com/en/resources/learning-center/basic-esp-operation.

[19] Babcock & Wilcox. Factors that affect ESP collection efficiency and ways to improve performance (2020, accessed 9 Dec 2020). https://www.babcock.com/en/resources/learning-center/factors-that-affect-esp-collection-efficiency-and-ways-to-improve-performance.

[20] Schorr, M. M. & Chalfin, J. Gas turbine NOx emissions approaching zero— is it Worth thePrice? (1999). https://www.ge.com/content/dam/gepower-pgdp/global/en_US/documents/technical/ger/ger-4172-gas-turbine-nox-emissions-approaching-zero-worth-price.pdf.

[21] United Nations Environment Programme (UNEP). The Global Sulphur Progress Tracker (2020, accessed 31 May 2020). https://www.unenvironment.org/resources/toolkits-manuals-and-guides/global-sulphur-progress-tracker.

[22] International Maritime Organization (IMO). International convention for the prevention of pollution from ships (MARPOL 73/78)—Annex VI—regulations for the prevention of air pollution from ships (2020).

[23] International Energy Agency (IEA). The future of hydrogen—seizing today’s opportunities. Paris (2019). 10.1787/1e0514c4-en.

[24] Worldsteel. Steel Statistical Yearbook 2019—Concise Version (2019). https://www.worldsteel.org/steel-by-topic/statistics/steel-statistical-yearbook.html.

[25] Daehn KE, Serrenho AC, Allwood JM (2017). How will copper contamination constrain future global steel recycling?. Environ. Sci. Technol..

[26] Remus, R., Aguado-Monsonet, M. A., Roudier, S. & Sancho, L. D. Best available techniques (BAT) reference document for iron and steel production (2013). 10.2791/97469.

[27] CONCAWE Air Quality Management Group’s Special Task Force on Integrated Pollution Prevention and Control. Refining BREF review—air emissions (2009). https://www.concawe.eu/publication/report-no-409/.

[28] World Business Council for Sustainable Developement (WBCSD). GNR Project Reporting CO2 2016 (2018, accessed 2 Oct 2018). https://www.wbcsdcement.org/GNR-2016/index.html.

[29] Steinmann Z, Hauck M, Schipper A, Huijbregts MAJ, Laurenzi I, Karuppiah R (2014). How to address data gaps in life cycle Inventories—a case study on CO2 emissions from coal electricity. Environ. Sci. Technol..

[30] Hauck M, Steinmann ZJN, Schipper AM, Gorrissen F, Venkatesh A, Huijbregts MAJ (2017). Estimating the greenhouse gas balance of individual gas-fired and oil-fired electricity plants on a global scale. J. Ind. Ecol..

[31] S &P Global. World electric power plants database (WEPP), version July 2020 (2020).

[32] Organisation for Economic Co-operation and Development (OECD). Developments in steelmaking capacity of non-OECD countries (2013). 10.1787/steel_non-oecd-2013-en-fr.

[33] Organisation for Economic Co-operation and Development (OECD). Capacity developments in the world steel industry (2016). http://www.oecd.org/sti/ind/Capacity-Developments-Steel-Industry.pdf.

[34] IndustryAbout. Worldwide Industrial Information 2018 (2018, accessed 2 Oct 2018). https://www.industryabout.com.

[35] Oil & Gas Journal. 2017 Worldwide Refining Survey (2017, accessed 17 Jun 2018). https://www.ogj.com/ogj-survey-downloads.

[36] Pro Global Media. Global Cement Directory 2017 (2017). https://www.globalcement.com/directory.

[37] European Environment Agency (EEA). European Pollutant Release and Transfer Register (E-PRTR) DK (2020).

[38] Raptis CE, Pfister S (2016). Global freshwater thermal emissions from steam-electric power plants with once-through cooling systems. Energy.

[39] Oberschelp C, Pfister S, Raptis CE, Hellweg S (2019). Global emission hotspots of coal power generation. Nat. Sustain..

[40] Ummel, K. CARMA revisited: An updated database of carbon dioxide emissions from power plants world wide. Washington DC. Working paper 304 (2012).

[41] energybase.ru - First-class communications and advertising in the Electric Power and Oil &Gas sectors of Russia and CIS. Energybase Energy Sites (2020, accessed 25 Jan 2020). https://energybase.ru/.

[42] Hofmann, F. & Hörsch,J. FRESNA powerplantmatching v0.4.1 (2019, accessed 5 Oct 2019). 10.5281/zenodo.3358985.

[43] Byers, L., Friedrich, J., Hennig, R., Kressig, A., Li, X. *et al*. Global power plant database— Version 1.2.0 (2019, accessed 5 Oct 2019). https://datasets.wri.org/dataset/globalpowerplantdatabase.

[44] Gupta, R. & Shankar, H. Global energy observatory (2018, accessed 11 Dec 2018). http://globalenergyobservatory.org/.

[45] International Energy Agency (IEA)—clean coal centre. Coal Power Atlas, London (2010, accessed 11 Oct 2017).

[46] U.S. Environmental Protection Agency (EPA). Emissions & Generation Resource Integrated Database (eGRID) 2018 v2 (2020).

[47] Environment and Climate Change Canada. National Pollutant Release Inventory (NPRI) (2019, accessed 27 Feb 2019). https://www.ec.gc.ca/inrp-npri/.

[48] Google LLC. Google Geocoding API. Mountain View, CA, US (2020). https://maps.googleapis.com.

[49] Australian Government—Department of the Environment and Energy. National Pollutant Inventory (NPI) (2020, accessed 15 Jul 2020). http://www.npi.gov.au/.

[50] Australian Government—Clean Energy Regulator. National greenhouse and energy reporting (NGER) data—electricity sector emissions and generation data 2016–17 (2019, accessed 2 Apr 2019). http://www.cleanenergyregulator.gov.au/NGER/National%20greenhouse%20and%20energy%20reporting%20data/electricity-sector-emissions-and-generation-data/electricity-sector-emissions-and-generation-data-2016-17.

[51] Environment and Climate Change Canada. Greenhouse gas reporting program (GHGRP)—facility greenhouse gas (GHG) data (2019, accessed 3 Oct 2019). https://open.canada.ca/data/en/dataset/a8ba14b7-7f23-462a-bdbb-83b0ef629823.

[52] U.S. Environmental Protection Agency (EPA). Air Emissions Inventories—2017 National Emissions Inventory (NEI) Data (2020, accessed 8 Jun 2020). https://www.epa.gov/air-emissions-inventories/2017-national-emissions-inventory-nei-data.

[53] U.S. Environmental Protection Agency (EPA). Greenhouse gas reporting program (GHGRP)—GHG reporting program data sets (2020, accessed 15 Jul 2020). https://www.epa.gov/ghgreporting/ghg-reporting-program-data-sets.

[54] China Electricity Council. 2015 National Thermal Power Unit Competition 100MW unit basic information table (in Chinese) (2016). http://kjfw.cec.org.cn.

[55] China Electricity Council. 2015 National Thermal Power Unit Competition 300MW unit basic information table (in Chinese) (2016). http://kjfw.cec.org.cn.

[56] China Electricity Council. 2016 National Thermal Power Unit Competition 600MW (including 1000MW) unit basic information table (in Chinese) (2017). http://kjfw.cec.org.cn.

[57] Central Electricity Authority (CEA). CO2 baseline database for the Indian power sector, v15.0 (2020, accessed 12 Jun 2020). http://cea.nic.in.

[58] U.S. Environmental Protection Agency (EPA). Emissions & Generation Resource Integrated Database (eGRID) 2018—PM2.5 supplement (draft) (2020).

[59] Eskom. CDM calculations—data requirements for calculating the Carbon Emission Factor (CEF) for the South African grid (2019, accessed 5 Apr 2019) . https://www.eskom.co.za/OurCompany/SustainableDevelopment/Pages/CDM_Calculations.aspx.

[60] Eskom. Atmospheric Emission License (EAL) reports (2020, accessed 26 Sep 2020). https://www.eskom.co.za/sites/publicdata/airquality/Pages/default.aspx.

[61] U.S. Energy Information Administration (EIA). Form EIA-860 detailed data 2016, 2019. https://www.eia.gov/electricity/data/eia860/. Accessed on 2019-04-08.

[62] U.S. Energy Information Administration (EIA). Form EIA-923 detailed data 2016 (2019, accessed 8 Apr 2019). https://www.eia.gov/electricity/data/eia923/.

[63] European Network of Transmission System Operators for Electricity (ENTSO-E). ENTSO-E Transparency Platform (2020, accessed 10 Feb 2020). https://transparency.entsoe.eu.

[64] Bundesnetzagentur. Power plant list version 2020-01-04 (in German) (2020, accessed 9 Jun 2020). https://www.bundesnetzagentur.de/DE/Sachgebiete/ElektrizitaetundGas/Unternehmen_Institutionen/Versorgungssicherheit/Erzeugungskapazitaeten/Kraftwerksliste/kraftwerksliste-node.html.

[65] Umweltbundesamt. Database ’Power Plants in Germany’ (in German) (2020, accessed 22 Feb 2020). https://www.umweltbundesamt.de/dokument/datenbank-kraftwerke-in-deutschland.

[66] U.S. Department of Energy (DOE). U.S. Department of energy combined heat and power installation database (CHPDB) (2020, accessed 1 Feb 2020). https://doe.icfwebservices.com/chpdb/.

[67] Dones, R., Bauer, C &, Röder, A. Part VI—coal—data v2.0 (in German) (2007). https://www.ecoinvent.org/.

[68] Raptis CE, Oberschelp C, Pfister S (2020). The greenhouse gas emissions, water consumption, and heat emissions of global steam-electric power production: A generating unit level analysis and database. Environ. Res. Lett..

[69] Fick SE, Hijmans RJ (2017). WorldClim 2: New 1-km spatial resolution climate surfaces for global land areas. Int. J. Clim..

[70] Wärtsilä. Combustion engine vs. gas turbine—part load efficiency and flexibility, (2019, accessed 22 Dec 2019). https://www.wartsila.com/energy/learn-more/technical-comparisons/combustion-engine-vs-gas-turbine-part-load-efficiency-and-flexibility.

[71] Brooks, F. J. GE Gas Turbine Performance Characteristics (2000). https://www.ge.com/content/dam/gepower-pgdp/global/en_US/documents/technical/ger/ger-3567h-ge-gas-turbine-performance-characteristics.pdf.

[72] U.S. Environmental Protection Agency (EPA)—Combined Heat and Power Partnership. Catalog of CHP Technologies (2017). https://www.epa.gov/chp/catalog-chp-technologies.

[73] Blair, N., DiOrio, N., Freeman, J., Gilman, P. & Janzou, S., *e*t al. NREL System Advisor Model (SAM) - Version 2017.9.5 (2017). https://www.nrel.gov/docs/fy18osti/70414.pdf.

[74] Perry RH, Green DW, Maloney JO (1997). Perry’s Chemical Engineers’ Handbook.

[75] European Environment Agency (EEA). EMEP/EEA air pollutant emission inventory guidebook 2019 (2019). https://www.eea.europa.eu/publications/emep-eea-guidebook-2019.

[76] Intergovernmental Panel on Climate Change (IPCC). 2006 IPCC guidelines for national greenhouse gas inventories—Volume 2—energy (2006). https://www.ipcc-nggip.iges.or.jp/public/2006gl/vol2.html.

[77] U.S. Environmental Protection Agency (EPA). CATC air pollution technology fact sheets (FS) and Technical Bulletins (TB) (2003, accessed 25 Sep 2020). https://www.epa.gov/catc/clean-air-technology-center-products#factsheets.

[78] GE Power. Air Quality Control Systems (AQCS) (2020, accessed 25 Sep 2020). https://www.ge.com/power/steam/aqcs.

[79] European Commission. Integrated pollution prevention and control- reference document on best available techniques for large combustion plants (2006). https://eippcb.jrc.ec.europa.eu/sites/default/files/2019-11/esb_bref_0706.pdf.

[80] Lecomte, T., de la Fuente, H., Ferreria, J., Neuwahl, F., Canova, M. *et al*. Best available techniques (BAT) reference document for large combustion plants (2017). 10.2760/949.

[81] Cheng T, Zhou X, Yang L, Wu H, Fan H (2020). Transformation and removal of ammonium sulfate aerosols and ammonia slip from selective catalytic reduction in wet flue gas desulfurization system. J. Environ. Sci..

[82] Leblanc, J. SkyscraperPage (2018, accessed 24 Jul 2018). http://skyscraperpage.com.

[83] Google LLC. Google Earth Pro v7.3.3.7786. (Mountain View, 2020). https://www.google.com/earth/.

[84] Ecoinvent centre. Ecoinvent v3.6 database—cut-off system model (Zurich, Switzerland, 2019). http://www.ecoinvent.org/.

[85] Intergovernmental Panel on Climate Change (IPCC). 2006 IPCC guidelines for national greenhouse gas inventories—volume 3—industrial processes and product use (2006). https://www.ipcc-nggip.iges.or.jp/public/2006gl/vol3.html.

[86] Oil & Gas Journal. 2015 world sulfur production (2015, accessed 17 Jun 2018). https://www.ogj.com/ogj-survey-downloads.

[87] International Energy Agency (IEA). IEA World Energy Statistics 2018. (2018, accessed 3 Dec 2018). 10.1787/data-00510-en.

[88] Environment Australia. Emission estimation technique manual for petroleum refining (1999). http://www.npi.gov.au/resource/emission-estimation-technique-manual-petroleum-refining.

[89] Barthe, P., Chaugny, M., Roudier, S. & Sancho, L. D. Best available techniques (BAT) reference document for the refining of mineral oil and gas (2015). 10.2791/010758.

[90] Elvidge CD, Zhizhin M, Baugh K, Hsu FC, Ghosh T (2015). Methods for global survey of natural gas flaring from visible infrared imaging radiometer suite data. Energies.

[91] Elvidge, C. D., Zhizhin, M., Baugh, K., Hsu, FC., & Ghosh, T. Global gas flaring observed from space (2012–2016) (accessed 10 Sep 2020)). https://eogdata.mines.edu/download_global_flare.html.

[92] U.S. Geological Survey (USGS). Minerals yearbook 2017—cement (Advance Release) (2020, accessed 11 Aug 2020). https://www.usgs.gov/centers/nmic/cement-statistics-and-information.

[93] Liu K, Wu Q, Wang L, Wang S, Liu T, Ding D, Tang Y, Li G, Tian H, Duan L, Wang X, Fu X, Feng X, Hao J (2019). Measure-specific effectiveness of air pollution control on China’s atmospheric mercury concentration and deposition during 2013–2017. Environ. Sci. Technol..

[94] Taiheiyo Cement Corporation. Report on environmental impact assessment for cement manufactured in countries around the world (2019). https://www.j-lca.sakura.ne.jp/english/lime/data/case1.pdf.

[95] Schorcht, F., Kourti, I., Scalet, B. M., Roudier, S., & Sancho, L. D. Best available techniques (BAT) reference document for the production of cement, lime and magnesium oxide (2013). https://eippcb.jrc.ec.europa.eu/sites/default/files/2019-11/CLM_Published_def_0.pdf.

[96] U.S. Environmental Protection Agency (EPA). Alternative control techniques document update—NOx emissions from New Cement Kilns (2007). https://www3.epa.gov/ttncatc1/dir1/cement_updt_1107.pdf.

[97] Oberschelp C, Pfister S, Hellweg S (2020). Globally regionalized monthly life cycle impact assessment of particulate matter. Environ. Sci. Technol..

[98] Cherubini F, Peters GP, Berntsen T, Strømman AH, Hertwich E (2011). CO2 emissions from biomass combustion for bioenergy: Atmospheric decay and contribution to global warming. GCB Bioenergy.

[99] R Core Team. R: A language and environment for statistical computing. Vienna, Austria (2019).

[100] Oberschelp, C., Pfister, S., Hellweg, S. Emissions and environmental impact dataset of global coal, gas, and oil power generation, steel mills, oil refineries and cement plants. Mendeley Data (2021). 10.17632/k2vgcm4bnk.1.

