# Supplementary material for: Global site-specific health impacts of fossil energy, steel mills, oil refineries and cement plants
Source: Sci Rep. 2023 Aug 22;13:13708. doi: 10.1038/s41598-023-38075-z (PMC10444750; doi:10.1038/s41598-023-38075-z)
Supplement: Supplementary file 1 — Supplementary Information 1. [file 41598_2023_38075_MOESM1_ESM.pdf]

Global site-specific health impacts of fossil energy, steel mills, oil refineries and cement plants - Supplementary Information

Christopher Oberschelp[a,b], **Stephan Pfister**[a], **and Stefanie Hellweg**[a,b]

[a]ETH Zürich, Institute of Environmental Engineering, John-von-Neumann-Weg 9, CH-8093 Zřich, Switzerland; [b]National Centre of Competence in Research (NCCR) Catalysis, ETH Zürich, CH-8093 Zürich, Switzerland

Supplementary materials and methods. This section provides further information on specific materials and methods that were not covered in the main paper.

Model overview. A visual overview of our emission and health impact model for power plants, steel mills, oil refineries and cement plants is shown in supplementary figure 1. In general, the basic facility data was first loaded and connected with several supplementary data sources before the respective activity data of each facility (electricity and heat for power plants, steel output for steel mills, crude oil inputs for oil refineries and clinker output for cement plants) in 2016 could be determined. This allowed to estimate the amounts of inputs of fuels and raw materials, which could then be used to estimate site-specific emissions. Where facility-level reported emission data was also available, these estimates could be used for disaggregation to individual emission sources per facility. As CO_2 emissions are typically closely related to fuel or raw material inputs through mass balances, we could use reported and disaggregated CO_2 emissions to improve earlier estimates of activity data and re-calculate the emissions for $PM_{2.5}$, SO_2, NO_x and NH_3 accordingly. The emission data was then supplemented by basic emission properties (like stack heights) to calculate the health impacts with the atmospheric model. Finally, their locations could be mapped.

Steam turbine thermodynamic data. Basic thermodynamic data like main steam pressures, main steam temperature and reheat steam temperatures for each generating unit were in most cases known from (1). Missing main steam pressures p_{main} [bar] for subcritical steam turbines were derived from a fit to the data points of figure 1 in (2):

$$p_{main} = 21.954 \cdot C_{unit}^{0.351} \qquad [1]$$

In this equation, C_{unit} [MW] is the electric generating capacity of the specific steam turbine. The lower limit of pressure p_{main} was set to 1 bar. In case the generating capacity of a combined cycle with a gas turbine and a steam turbine is reported as a sum, we generally assume that two thirds of the generating capacity belong to the gas turbine and the remaining one third to the steam turbine (based on the disaggregated capacities of combined cycle units reported in (1)). The pressure of steam turbines from combined cycle power plants with internal combustion engines is relatively independent of its capacity and we thus assume it to be 15 bar in case of data gaps (based on the median reported in (1)).

Missing main steam temperatures T_{main} [°C] for subcritical steam turbines were then calculated as:

$$T_{main} = 542 \cdot (1 - exp(-0.0392 \cdot p_{main})) \qquad [2]$$

This equation has also been obtained from (2). The lower limit of temperature T_{main} was set to 100 °C. The main steam temperatures of steam turbines from combined cycle power plants with internal combustion engines were a special case as they were set to the median of 345 °C from (1). Based on average data contained in (1), the main steam pressure and temperature of supercritical steam turbines were assumed as 245 bar and 550 °C, and for ultrasupercritical steam turbines as 260 bar and 595 °C, respectively. Following the procedure from (2), all combinations of steam temperatures and pressures were checked to see whether the water would actually be steam at that conditions. Where that was not the case, the steam temperature was increased with equation 2.

As most steam turbine units with electric generating capacities larger than 100 MW have one reheat stage, we also made that basic assumption for generating units missing main steam temperatures. The mean ratio of reheat temperatures to main steam temperatures in (1) is 1.002 (in [°C/°C]), which is also what we assumed for unknown reheat temperatures.

The classification of main steam conditions in the categories subcritical, supercritical and ultrasupercritical was available for most units, but for a small number of data gaps (<60), the classifications were added based on an analysis of the existing data in (1). For these, steam turbines with electric generating capacities below 300 MW as well as any steam turbines in combined cycle power plants were classified as subcritical, those greater than or equal to 300 MW but below 900 MW as supercritical and the rest as ultrasupercritical.

To determine the lowest temperature of the Rankine cycles of each steam turbine, the cooling system had to be known. Most cooling systems were already classified (as discussed in (3)) and the remaining ones were filled in based on the most common technology. An analysis of the reported data showed that once-through cooling is the most common technology for old steam turbine power plants, while newer ones mostly use wet tower cooling. Thus, the missing cooling systems for all units constructed before 1990 or with unknown construction date were assumed to use once-through cooling, while all newer units were assumed to use wet tower cooling.

The lowest temperature of the Rankine cycle T_{min} [°C] per steam turbine was then derived as combination of the cooling system and the temperature of the surrounding environment. For dry air cooling, the average of the site-specific monthly air temperatures from (4) (down to a minimum of +5 °C) was used, while water temperatures for wet cooling systems were calculated from the correlation of air and water temperatures presented in (5) (also down to a minimum of +5 °C). A temperature difference of +10 °C was added to the respective cooling temperature as driving temperature gradient for heat transfer (based on (3)). Wet bulb temperatures for wet tower

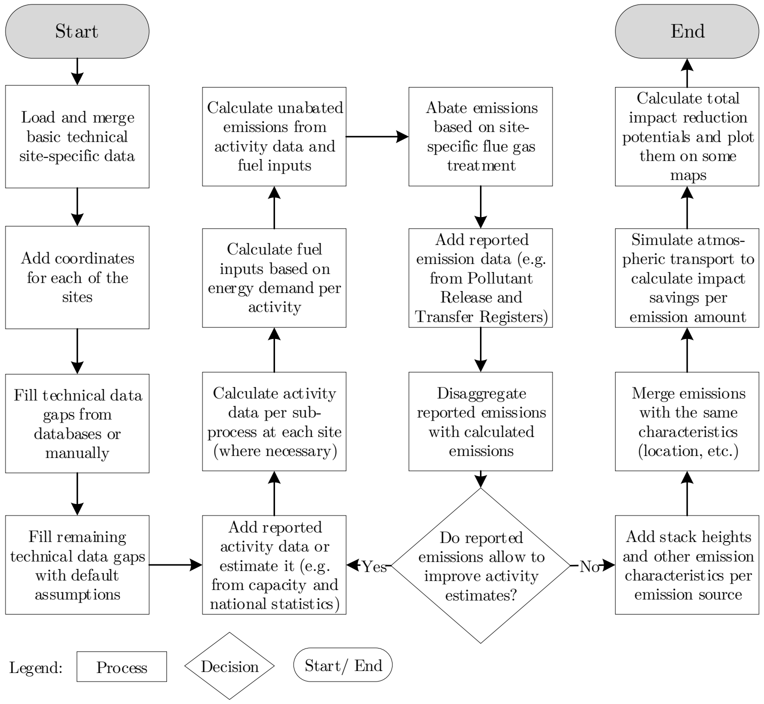

Supplementary figure 1. *General overview of the site-specific emission and impact calculation procedure.*

cooling were not further distinguished considering the limited influence of the cooling temperature on the overall efficiencies (3) and the uncertainties in the other data for calculating Rankine cycle efficiencies.

Boiler classification. The classification of boiler types was necessary for filling in missing boiler efficiencies η_b [-] as described in the main manuscript and for assigning emission factors. By default, oil and gas-fired boilers were assumed to be dry bottom boilers as these are most common according to (6). In case of coal-fired boilers, different fuel types and sizes were distinguished. Anthracite and lignite boilers up to an electric gross generating capacity of 10 MW were assumed to be fixed bed boilers, those larger than 10 MW but less than or equal to 300 MW as fluidized bed boilers and the rest as dry bottom boilers. Bituminous and subbituminous coal boilers with electric gross generating capacities below 50 MW were assumed to be fixed bed boilers and the rest dry bottom boilers. These size limits for the default assumptions were derived from the reported data in (1, 6). The coal fuel types were based on the primary fuel presented in (1), while gaps per unit were filled from (7) where possible and else assuming bituminous coal, which is by far the most common coal type in power generation globally (8).

Steam power plant efficiencies. The calculation procedure for steam turbine efficiencies follows the basic framework from (2, 3, 7) and makes several updates to it. The gross electricity generation $\dot{W}_{el,gross}$ [MWh/a] was calculated from the net electricity generation $\dot{W}_{el,net}$ [MWh/a] and the net-gross efficiency η_{ng} [-]:

$$\dot{W}_{el,gross} = \frac{\dot{W}_{el,net}}{\eta_{ng}} \quad [3]$$

The net-gross efficiency was either known for the specific unit or it was for coal power plants derived from a regression that was fit to the reported data from (9–11):

$$\eta_{ng} = 0.947 - 0.05 \cdot exp(-0.004 \cdot C_{unit}) \quad [4]$$

In case of Indian lignite and non-lignite coal power plants, the guidelines of the Indian Central Electricity Authority (12) as presented in supplementary tables 15 and 16 were used for linear interpolation. For other types of fuels, the net-gross efficiencies were selected as shown in supplementary table 14. The average fuel input $\dot{m}_{fuel}$ [kg/s] per electricity generation was then calculated with equation 4 in the SI of (7) including the modifications to the boiler efficiencies η_b [-] and lower heating values LHV [MJ/kg] described in the main manuscript. Mechanical turbine efficiencies η_t [-] were selected as in (7). Generator efficiencies η_g [-] were derived by linear interpolation with unit capacities C_{unit} and the efficiencies according to supplier data shown in supplementary table 13.

Rankine cycle efficiencies were generally calculated by a procedure derived from the approach of (2) with several central changes:

- The calculation procedure for the enthalpy of all pumps was changed so that their isentropic efficiencies could be included in the same way as for steam turbines. Thus, for example, the pump equation for the enthalpy in section S2.2.1 of (2) was changed to

$$h_2 = h_1 + \frac{h_{2s} - h_1}{\eta_{pump}}, \quad [5]$$

in which h_1 [kJ/kg] is the specific enthalpy in front of the pump, h_2 [kJ/kg] the specific enthalpy behind the pump, h_{2s} [kJ/kg] the isentropic specific enthalpy behind the pump and η_{pump} [-] the isentropic pump efficiency. The

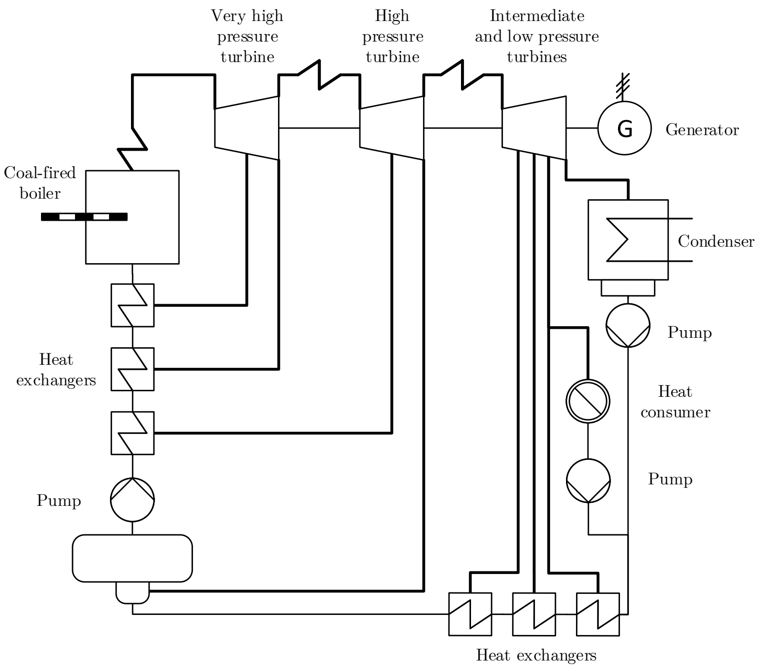

Supplementary figure 2. *Simplified heat flow diagram for an extraction condensing supercritical coal power plant with dual reheat and co-generation of heat and power as modeled by this work.*

value for η_{pump} was chosen in accordance with (2) and h_{2s} was calculated with the pressure behind the pump p_2 [bar] and the specific entropy in front of the pump s_1 [kJ/kg/K] based on IAPWS-95 guidelines (13). The specific entropy s_1 was, in turn, calculated according to IAPWS-95 guidelines from the known temperature in front of the pump T_1 [K] and under the assumption that the water was saturated. All other pump equations were changed accordingly.

- The isentropic steam turbine efficiencies η_t [-] depend on pressure ratios so steam turbines with higher pressure ratio tend to have higher isentropic efficiencies (14). As the lower pressure of condensing steam turbines is near vacuum for all these turbines, we use the maximum steam pressure to interpolate isentropic full load turbine efficiencies linearly based on typical manufacturer data as shown in supplementary table 22.
- At part-load operation, the isentropic steam turbine efficiency decreases. We thus adjusted the isentropic full load turbine efficiencies with a factor calculated by the procedure from (15) for the assumed average load.
- All steam turbines except for the most simple ones at very small electric generating capacities use regeneration to improve their electric efficiency. In the work of (2), in contrast, none of the simple Rankine cycles without co-generation were modeled with regeneration. Thus, we introduced regeneration even for all simple Rankine cycle steam turbines with electric generating capacities greater than or equal to 10 MW. This limit was chosen based on manufacturer data. All equation systems for simple Rankine cycles in (2) were extended in accordance with the examples in there.
- Rankine cycle steam turbines use a large amount of extractions (up to about 10) for regeneration while (2) assume usually just one (co-generation or single reheat cycles) or maximal two extractions (for dual reheat cycles), which underestimates electric efficiencies. The exact amounts and thermodynamic conditions of extractions are turbine-specific, and each additional extraction added to the model of (2) improves the electric efficiency less than adding the previous one. After comparing several modeling options, we decided to assume three extractions on the lowest turbine stage, and two extractions for each reheated turbine stage (supplementary figure 2). Thus, a simple cycle had three extractions, a single reheat cycle five extractions and a dual reheat cycle seven extractions. More extractions would have only minor influence on the thermodynamic efficiency of the cycle, but considering the uncertainties due to main steam conditions and cooling temperatures as well as lack of data on the exact extraction amounts for each turbine, their addition was assumed unnecessary. The extraction for co-generation was assumed to be at the lowest pressure level of all extractions. All equation systems were extended in line with the procedure from (2) and the exact pressure levels were chosen by the optimization procedure described therein.
- In the work of (2), the heat and electricity outputs of co-generation were implicitly defined through fixed ratios of useful heat output per fuel heat input. In reality, though, heat-to-power (HTP [-]) ratios reflect the directly measurable supply of heat and power of each generating unit, while the heat inputs vary over time due to inhomogeneous fuel properties and are thus more difficult to determine. As a consequence, the HTP ratios are frequently known per unit, which is why we reformulated the co-generation models of (2) to contain HTP ratios as input parameter. In the simple Rankine cycle with co-generation case (section S2.2.4 of (2)), the HTP would therefore be defined as:

$$HTP = \frac{f_c \cdot (h_6 - h_4)}{1 \cdot (h_4 - h_3) + (1 - f_c - f) \cdot (h_5 - h_4)} \quad [6]$$

All specific enthalpies h [kJ/kg] and the mass fractions f_c [kg/kg] and f [kg/kg] are described in (2). The definition of the HTP ratio would then replace the definition based on the total heat input $f_c \cdot (h_4 - h_6) = 0.45 \cdot (h_3 - h_7)$ and the equation system would be solved for η_{cycle} to replace equation *d1* in (2). This change has been implemented for all extraction condensing Rankine cycles accordingly.

- A Rankine cycle with dual reheat, regeneration and co-generation did not exist in the work of (2) for 2012. In 2016, several power plants use this type of Rankine cycle and thus, the corresponding equation system was developed for this case based on the other cases in (2) and the adjustments made in the present work. The basic heat flow diagram for dual reheat, regeneration and co-generation is shown in supplementary figure 2 and an example for the thermodynamic properties in supplementary figure 3.
- Back-pressure steam turbines do not follow the same Rankine cycles as extraction condensing turbines described in (2) since they lack the condensing step at vacuum, but they were not modeled separately in (2). At the same time, their HTP ratio is typically substantially higher than for extraction condensing turbines (14), which leads to high heat utilization. Thus, we approximated their electric efficiency η_{el} [-] with the following equation that yields very similar results to the cases presented in (14):

$$\eta_{el} \approx \frac{1}{HTP + 1} \quad [7]$$

- As back-pressure steam turbines co-generate heat in the form of steam, we assume a minimal temperature of 100 °C and a minimal pressure of 1 bar for the released heat. In consequence, the range of feasible HTP ratios is reduced. Where earlier estimates of HTP ratios (for example based on statistics in (8)) for individual steam turbines were technically infeasible, we adjusted them to the closest limit of the feasible HTP ratio range.

Once the efficiencies of all steam generating units were calculated, their maximum rated thermal input $\dot{W}_{th,in,max}$ [MWh/a] could be determined with the electric gross generating capacities C_{unit} from (1):

$$\dot{W}_{th,in,max} = \frac{C_{unit} \cdot 8760\,\frac{\mathrm{h}}{\mathrm{a}}}{\eta_{el} \cdot \eta_b \cdot \eta_g \cdot \eta_t} \quad [8]$$

In this equation, η_{el} [-] denotes the thermodynamic efficiency of the steam cycle, η_b [-] the efficiency of the boiler, η_g [-] the generator efficiency and η_t [-] the mechanical efficiency of the turbine. The maximum rated thermal input was then available to select appropriate emission factors as described below.

Gas turbine and internal combustion engine efficiencies. Gas turbines and internal combustion engines lacked basic thermodynamic data to calculate electric efficiencies for each generating unit, so gross electric efficiencies $\eta_{el,gross}$ [-] for specific gas turbines and combustion engines were obtained from technical data sheets as described in the main manuscript. Where the type of gas turbine or combustion engine of a generating unit was unknown, the efficiencies were approximated by gross electric capacity-based correlations. These regressions were derived from fits to the known efficiencies that were collected from the data sources listed in supplementary table 26. Thus, the gas turbine efficiencies could be calculated as:

$$\eta_{el,gross} = 2.65 - 2.79 \cdot (C_{unit} + 94.7)^{-0.0363} \quad [9]$$

An upper limit for the gas turbine capacities C_{unit} was set to 600 MW as this is the size of largest currently available units. The resulting efficiencies thus ranged from about 30 % to 45 %. For internal combustion engines, the efficiencies as derived from the data sources listed in supplementary table 26 were calculated as:

$$\eta_{el,gross} = 0.480 - 1.41 \cdot (C_{unit} + 4.22)^{-1.61} \quad [10]$$

The upper limit for internal combustion engine capacities per unit was set to 20 MW and so the resulting efficiencies ranged from about 32 % to 47 %. In case capacities were reported as sum of several units in (1), the average unit capacity was used for each of the individual units. Gross electric efficiencies were adapted to site-specific conditions based on reported data. Gas turbine efficiency derating for part-load operation was linearly interpolated with the data in supplementary table 17 (16), derating based on humidity with the data in supplementary table 18 (17) and derating based on inlet temperature with the data in supplementary table 19 (17). The specific humidity of air for use with the humidity derating data was calculated from the local air temperature and relative humidity with the Magnus equation. Gas turbine efficiencies were reduced by 4.05 % in case they were burning oil instead of gas based on (17). Internal combustion engine part-load derating was calculated with the data in supplementary table 20 based on (16). The derating of internal combustion engines based on inlet air pressure due to the altitude and the inlet air temperature were used from (14). Their maximum rated thermal input was determined with the electric gross generating capacities C_{unit} from (1):

$$\dot{W}_{th,in,max} = \frac{C_{unit} \cdot 8760\,\frac{\mathrm{h}}{\mathrm{a}}}{\eta_{el,gross}} \quad [11]$$

This allowed to select appropriate emission factors for each of the units (details below). Finally, net-gross efficiencies were added from supplementary table 14 so that fuel inputs as a function of the amount of electricity generated could be calculated:

$$\dot{m}_{fuel} = \frac{\dot{W}_{el,net}}{\eta_{ng} \cdot \eta_{el,gross}} \cdot \frac{1}{LHV} \quad [12]$$

Fuel inputs for gas turbines and internal combustion engines in combination with emission factors were thus available to calculate fuel-related emissions for these units as described below.

Computational structure of inventories. The inputs and outputs of power generating units were directly associated with their emissions. In case of steel mills, oil refineries and cement plants, however, each emitting site consists of several sub-processes that depend on each other. Thus, more complex equation systems needed to be set up to calculate inputs

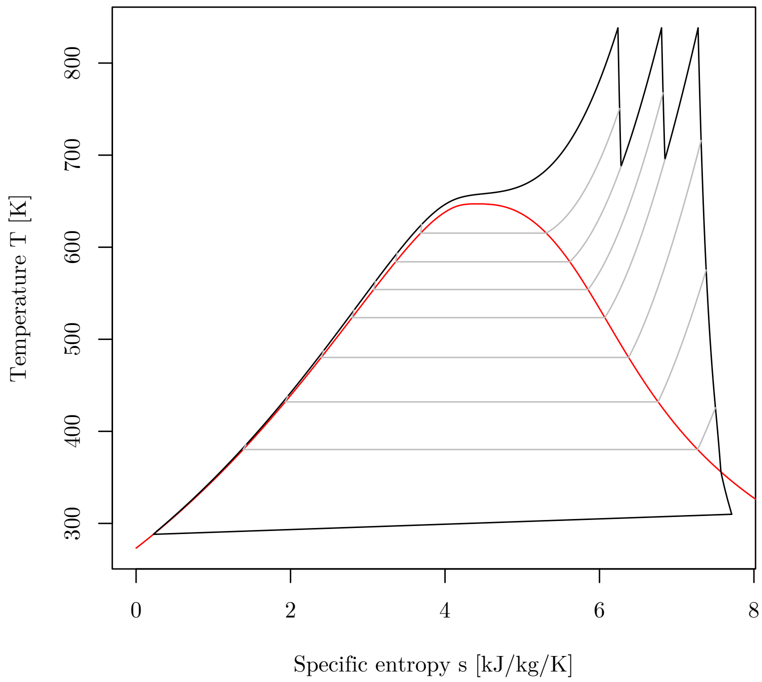

Supplementary figure 3. *Temperature-entropy diagram (T-s diagram) of the coal power plant with dual reheat and co-generation of power and heat as depicted in supplementary figure 2. The thermodynamic conditions of the Rankine cycle are shown in black and the conditions of the seven extractions in gray. The limits of liquid phase and gas phase are shown in red.*

and outputs. It is convenient to use the matrix notation of regionalized LCA to provide some basic structure to that. Several such notations have been defined (for example (18–20)), which differ in details and mathematically lead to the same results. We use the notation of (20) with a technosphere matrix **A** that contains the interrelations of the individual sub-processes at a site and a biosphere matrix **B** with the emissions of each sub-process to the environment. As we do not have data about exchanges between different sites, we set up individual site-specific matrices **A** and **B** for every site separately. Default technosphere matrices **A** for steel mills, oil refineries and cement plants before replacing individual flows with site-specific data are presented in supplementary tables 6, 7 and 8, respectively, while the corresponding default biosphere matrices **B** are given in supplementary tables 9, 10 and 11. Site-specific modifications of these matrices are listed in supplementary table 23.

Fuel properties. Fuel properties like sulfur and ash content in this work have been obtained for coal power plants from the earlier work of (7), while for units with missing data the default values from (21) were used. A few additional modifications were made to update the coal data to 2016 conditions. The ash content of Chinese coal was set to 21.6 wt.-% based on the power plant design data reported in (9–11). The Indian ash content of coal was set to 35 wt.-% based on (22). Ash content outliers above 40 wt.-% were replaced with the default values from (21). In case of coal sulfur content, an upper limit of 0.86 kg kJ^{-1} based on (23) was set for coal power plants that use coal washing or low-sulfur coal as only measures to control SO_2 emissions. And sulfur content outliers higher than 5 wt.-% were finally also replaced with the default values from (21). In case of diesel, the 2016 sulfur limits from (24) were used. As comparable data was unavailable for various types of fuel oils, we filled this gap with default assumptions. Thus, the sulfur content of light or distillate fuel oils in developed countries was set to 0.1 wt.-%, in developing countries to 1 wt.-% and in least developed countries to 2 wt.-%. For heavy or residual fuel oils as well as crude oil, we assumed the sulfur contents to be 1.0 wt.-% in developed countries, 2.5 wt.-% in developing countries and 3.5 wt.-% in least developed countries. The classification of countries into these three categories followed (25, 26). Natural gas properties were assumed to match the default values from (21).

Default flue gas treatment. Several default assumptions were made to fill data gaps in terms of flue gas treatment. Coal power plant flue gas treatment data was generally filled as described in (7) with minor additions to account for new developments. Thus, the default NO_x control for Chinese coal power plants with electric generating capacities larger than or equal to 300 MW were set to a combination of low NO_x burners and selective catalytic reduction based on the unit data from (9–11). In India, low NO_x burners were assumed for new coal power plants that were constructed from 2015 onwards as this is the year that NO_x regulation for these plants was introduced. The default flue gas treatment assumptions for gas and oil power plants generally followed the documentation of (27) and the data in (1), while it was also assumed that gas or oil power plants were not regulated more strictly than coal power plants. Thus, it was assumed that gas turbines from before 2010 or in India from 2015 onwards used water injection for NO_x control while gas or oil-fired steam turbines used low NO_x burners. Gas turbines from 2010 or newer, in contrast, were assumed to use dry low NO_x combustion systems, which was also added in case they were reported to use selective catalytic reduction. For heavy fuel oil, residual fuel oil or crude oil-burning steam turbines, it was additionally assumed

that they used at least some mechanical flue gas treatment device to reduce primary PM emissions. For steel mills, oil refineries and cement plants, the default flue gas treatment in case of data gaps for individual sites was selected as listed in supplementary table 23.

Flue gas treatment removal rates. The removal rates for flue gas treatment of power plants η_{FGT} [-] were based on non-linear functions so that higher unabated pollutant concentrations c_{in} (in [g/Nm^3] for $PM_{2.5}$ and in [ppmvd] for SO_2 and NO_x) also lead to higher removal rates, while lower pollutants concentration lead to lower removal rates. At the same time, the higher unabated pollutant loads were supposed to lead to higher final emissions when comparing the same flue gas treatment at lower load. Thus, typical load ranges from $c_{in,min}$ to $c_{in,max}$ (also in [g/Nm^3] or [ppmvd]) and removal efficiency ranges from $\eta_{\mathrm{FGT},min}$ to $\eta_{\mathrm{FGT}max}$ [-] were obtained or estimated for the individual flue gas treatment types as shown in supplementary table 3 based on (28–30). Unabated pollutant concentrations c_{in} were then calculated with the excess air factors in supplementary table 3 based on (31) and supplier data assuming an ideal gas. The flue gas treatment removal rate η_{FGT} of each unit was calculated with

$$\eta_{\mathrm{FGT}} = 1 - \frac{\alpha}{\beta + c_{in}} \qquad [13]$$

where the parameters α and β were

$$\alpha = (1 - \eta_{\mathrm{FGT},min}) \cdot (c_{in,min} + \beta) \qquad [14]$$

and

$$\beta = \frac{(1 - \eta_{\mathrm{FGT},max}) \cdot c_{in,max} - (1 - \eta_{\mathrm{FGT},min}) \cdot c_{in,min}}{(\eta_{\mathrm{FGT},max} - \eta_{\mathrm{FGT},min})}. \qquad [15]$$

At unabated pollutant loads below the typical range, the minimal removal rate $\eta_{\mathrm{FGT},min}$ was assumed, and above the typical range the maximal removal rate $\eta_{\mathrm{FGT},max}$. Combinations of flue gas treatment technologies were calculated sequentially, including for combined cycle power plants, in which for example the abated exhaust gases of several gas turbines fed steam turbine HRSGs with additional flue gas treatment. Then, the abated pollutant concentration c_{out} (also in [g/Nm^3] for $PM_{2.5}$ and in [ppmvd] for SO_2 and NO_x) was calculated:

$$c_{out} = c_{in} \cdot (1 - \eta_{FGT}) \qquad [16]$$

Finally, the abated pollutant concentration c_{out} could be used to calculate the pollutant emissions assuming an ideal gas. Flue gas treatment for steel mills, oil refineries and cement plants was calculated using fixed removal rates as detailed in supplementary table 23 with the flue gas treatment technology data in supplementary tables 4 and 5.

Emission properties. Besides the emission flow rates and stack heights for each emission source, the atmospheric model from (32) required corresponding emission source diameters, emission velocities and emission temperatures as input. The emission source diameters have been interpolated linearly from the emission height based on the data in (32), thus assuming an emission diameter of 0.15 m for emissions at 2 m height and below, a diameter of 1 m for emissions at 25 m height, and a diameter of 6.4 m for emissions at 100 m height and above. The emission velocity was set to 20 m s^{-1} based on (32), which is also in good agreement with (6), and the emission temperature was set to 423.15 K based on the reported values from (6).

Pollutant terminology. The pollutant terminology in this work is identical to the one used in (32). Thus, the term primary $PM_{2.5}$ comprises both the filterable as well as the condensable fraction of $PM_{2.5}$ directly emitted by a pollution source. Secondary $PM_{2.5}$, on the other hand, comprises the $PM_{2.5}$ species that are not emitted as $PM_{2.5}$ but transformed chemically from other pollutants into filterable or condensable $PM_{2.5}$ in the atmospheric. NO_x comprises both NO and NO_2, and is expressed in NO_2-equivalents. Not all data sources used in this work follow this naming convention and they regularly do not even specify their terminology at all. Thus, undocumented pollutant terminology specifications in other work might cause some inconsistencies.

Supplementary results and discussion. Additional results and their discussion are presented below.

Model validation. The emission model for power plants is an extension of the earlier work from (7) and the validation of fossil power plant emissions is discussed there. A comparison of modeled and reported emissions for steel mills, oil refineries and cement plants is presented in supplementary figure 4 for validation purposes. The results show that overall CO_2 emissions for each of emission source types match well, even though the differences are somewhat larger than for fossil power generation. Three main reasons for that can be observed. First, the 2016 activity data for the individual sites is unknown and needs to be estimated from national statistics. Steel mills, oil refineries and cement plants all aim at high production amounts to utilize the existing equipment to the extent possible, but both technical and economical factors may lead to higher or lower than average utilization. Second, there is a certain flexibility in the fuel types used at each site, for which we also only have limited site-specific data. As the typical CO_2 emission intensities for fossil fuels may range from 260 kg GJ^{-1} for blast furnace gas to 44 kg GJ^{-1} for coke oven gas (33), this also explains some of the deviations of the absolute CO_2 emissions. And finally, the reported emissions may in some cases also include other on-site activities with CO_2 emissions that are not included in our model and thus increase the reported emissions above the calculated emissions. In case of steel-making, this could for example be on-site power generation that is not reported separately and thus contributes to the total CO_2 emissions of the site. Such deviations are most pronounced for some large-scale oil refineries, possibly because a considerable share of downstream processing from basic refinery products into more valuable products such as some petrochemicals is taking place at the refinery sites and thus is also included in the emission reporting. Since we lack data about other on-site activities, we cannot include them into the models at the current time, but this would be worth some future investigation. The overall influence on CO_2 emissions is relatively small though, as the most energy-consuming and CO_2-emitting parts of each site seems to be covered well.

For $PM_{2.5}$ emissions, supplementary figure 4 shows that data reporting is less common than for CO_2 emission and the

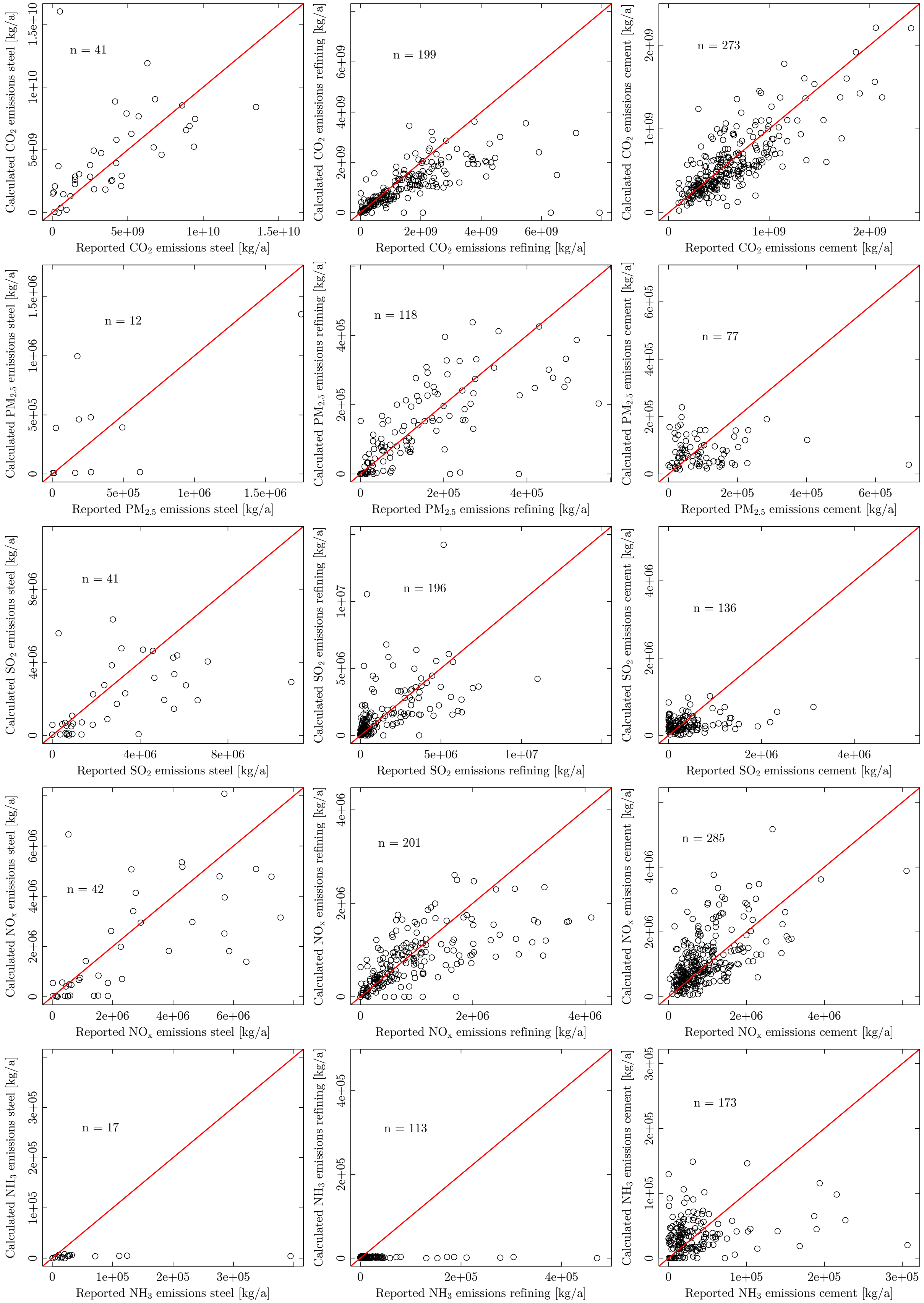

Supplementary figure 4. *Comparison of reported and modeled annual CO_2, $PM_{2.5}$, SO_2, NO_x and NH_3 emissions for steel mills, oil refineries and cement plants.*

validation of the emission model, especially in the case of steel mills, is more difficult. The lack of reported $PM_{2.5}$ in Europe, where reporting is only available for PM_{10}, is one of the main contributors to this. The deviations between reported and calculated emissions are found higher than for CO_2, which has already been observed for fossil power generation by (7). The reasons lie in the fact that $PM_{2.5}$ emissions cannot be calculated by simple mass-balancing as in case of CO_2 but they are subject to more complex formation and abatement mechanisms that in some cases cannot be measured easily (like for example in case of diffuse emissions from material handling or in case of flaring, where a share of the particles are formed in the open air after the burning substances have left the opening of the flare). More deviations may be due to reporting differences as it is not always clear, which types of emission sources at each site have been included, and what exactly is meant with $PM_{2.5}$ (e.g. condensable or filterable PM). Especially few data points are available for steel-making, where the few data points available may indicate a high variability but are insufficient to further investigate the causes. For oil refining, in contrast, there is much more $PM_{2.5}$ emission data available, which indicates an overall good match between calculated and reported data, especially considering the range of emission sources and the different types of flue gas treatment in use. In case of cement-making, there are also more data points available than for steel-making and these show generally decent agreement between measured and reported values but there are also considerable amounts of outliers in both directions. The reasons are not entirely clear, but the outliers may in part be explained by the fact that most of the cement-plant data points are from the US, where site-specific particle filter technology data was not available for about half of the cement plants and thus had to be filled with default assumptions. In addition, the US $PM_{2.5}$ emissions had to be extrapolated from 2017 NEI data (34) and are thus also subject to extrapolation-related deviations.

The SO_2 emissions show high variability across the three types of emission sources and underline the value of emission measurements and public reporting of the results. In particular, the SO_2 emissions depend on fuel qualities that are not reported and have also not been investigated in detail (in contrast to the calculations for coal power supply and combustion in (7)). In that light, especially the refinery SO_2 emission estimates show good agreement between model and measurements considering the variety of sources (fuel supply, flaring, sulfur production, catalyst regeneration) and the uncertainties in the modeling approach. Further reductions of deviations might be possible with estimates about the sulfur content in the crude oil (as implemented in the refinery model used by (35)), but this would then require a considerably different modeling approach. For cement-related SO_2 emissions, the main trends match well but individual deviations remain high. Here, the quality of the raw material inputs other than the fuels may be an important factor, as the lime content of the raw inputs may lead to the formation of gypsum, which effectively abates part of the potential SO_2 emissions. Composition data about cement plant raw material inputs were not available, so this mechanism could not be covered in detail and the uncertainties in the input data hence may be responsible for the deviations between modeled and reported results.

For NO_x emissions, the combustion conditions are the main driver of emissions and so the nitrogen contents of fuels play a smaller role in explaining the observed differences between calculated and reported emissions. Also in this case, steel mill data is scarce with high variability in the results and unclear reasons. The refinery results, in contrast, match overall very well, which also indicates that data gaps in terms of pre-combustion and post-combustion emission reduction measures at boilers and furnaces have been filled sufficiently. To which degree reported refinery NO_x emissions reflect the true variability of difficult-to-measure emissions from the flare, is currently not clear. A small number of about six NO_x emission estimates that are much higher than we calculated may either be due to other on-site NO_x emission sources not covered by our model or by the lack of NO_x abatement equipment, which we assume by default. The NO_x emissions for cement plants also fit very well, which asserts us that our approach for dealing with recent NO_x emission abatement techniques that were not covered in (28) works as intended and that the lack of considering abatement techniques in (36) leads to a substantial overestimation of current cement production NO_x emissions.

The NH_3 emission comparison for steel mills and oil refineries reflects the general lack of sufficient emissions factors or emission calculation guidelines for these processes. Thus, we underestimate the NH_3 emissions of this type of facility with our work. Other contributors may be additional on-site activities causing NH_3 emissions, but we lack appropriate activity or capacity data to include them. Nevertheless, the reported NH_3 emissions per steel mill or oil refinery seem to be in a similar range compared to cement-making, while there are many more cement plants than steel mills or oil refineries, and NH_3-related health impacts from cement-making were found to be already comparably small. Thus, the underestimation may not be a critical problem even though further work on this issue would be desirable. For cement-making, the reported and modeled NH_3 emissions lie in the same range, but show very high differences between both in individual cases. The amount of ammonia slip from SNCR operation depends much on the injected NH_3 amount, which is directly related to the desired NO_x reduction. Modern clinker kilns take a reduced formation of NO_x into account by optimized kilns designs, so it is likely that different amounts of NH_3 need to be injected to reach the emission limits based on the individual kiln. Our model underestimates such effects as we do not have detailed data on this available.

Overall, the emission estimates for steel mills, oil refineries and cement plants seem to match the trends in the reported data quite well but of course may show major deviations in individual cases. For a global-scale inventory, this is an expected outcome and matches the pattern for fossil power generation in (7). As many sites were covered with the emission inventory and no major systematic deviations were observed, while fate and effect per amount of pollutant may differ by several orders of magnitude (32), it can be assumed that the deviations between measured and reported emissions have limited influence on results and conclusions. The lack of reported data for steel mills remains to be a challenge. This is especially so for steel mills, but also for other types of facilities, in developing and least developed countries, where emission standards, equipment and operational practices may or may not differ from developed countries. In addition, emissions may fluctuate

over time, especially when starting up the equipment and this might also not be fully captured where emission measurements were unavailable. Uncertainties regarding emission intensities based on such approaches may however be small in comparison to uncertainties due to the complex assumptions in the health impact assessment.

Other airborne pollutants. Methane (CH_4) emissions may be relevant contributor to health impacts from climate change. The CO_2 covered within this study mostly arise from combustion processes, which have low emission rates for methane in comparison for CO_2. For example, the typical emission factors of natural gas (33) suggest emission intensities that are about a factor of 50 000 lower than for CO_2. Similar ratios apply to other fossil fuels. Even with a characterization factor of methane that is 28 times higher than for CO_2, the contribution of methane emissions to the total health impacts would be several orders of magnitude smaller than for CO_2 and is therefore omitted from the assessment.

Other relevant emissions for the formation of secondary $PM_{2.5}$ may include volatile organic compounds (VOCs). These, however, are currently not covered by any similar health impact assessment method as the one that is used for the other types of secondary $PM_{2.5}$ considered within this study. Refineries might be a relevant source for VOCs, but the lack of impact assessment methods makes it impossible to cover them within the present study.

Emissions and health impact reduction potentials by country. The data on emissions and health impact reduction potentials by country shown in supplementary figure 5 is complementary to figure 1 in the main manuscript in the way that it shows the same data from a country perspective instead of a source type perspective. The two drivers for health impacts globally that stand out most come from the high CO_2 emissions in China and the high SO_2 emissions in India. The major cause for both is coal power generation in the respective countries. Thus, the visualization of results in supplementary figure 5 underlines the findings discussed in the main manuscript and is also in excellent agreement with the more detailed regional distribution of PM-related health impact saving potentials shown in figure 2 of the main manuscript. Overall, the Group of Twenty (G20) is responsible for more than 75 % of potential health impacts from PM and for more than 85 % in case of CO_2 for the covered emission sources. Priorities lie with China, the United States, India, and the European Union, followed by Japan and Russia. The global pattern is a clear representation of how the industrialized nations such as the US or the EU have successively outsourced their energy-intensive industries to developing countries such as China or India (37), which are now in turn looking for ways to outsource their energy-intensive industries further as they are on the verge of becoming industrialized countries themselves (for example in the form of the *Belt and Road Initiative* in China). Still, the emissions of pollutants with global health impacts have remained high also in the industrialized countries, but the pollution with local and regional health impacts is, in contrast, much smaller due to stricter local regulation, lower population densities and frequently also more advanced technology.

Comparison with other work. The overall emission patterns in this work match well with other studies (38–40), but detailed comparisons per industrial site are generally not possible due to the unavailability of high-resolution data on a global scale. Likewise, the global health impact pattern with hotspots in Asia, Europe and North America matches well with other studies that only link pollutant exposure with health impacts (41), but a more in-depth comparison is also there not possible due to differences in scope and level of detail. In addition, the patterns of health impacts per amount of emissions are generally similar compared to (32), where strongly simplified emission heights from coal power plants and smaller region for high-resolution pollutant fate modeling was used, but especially the health impact contributions from SO_2 in the present study are higher as delayed chemical reactions and extremely high stacks in regions such as India or China distribute the pollutants over large areas. Thus, SO_2 health impacts show a wider regional spread than for example primary $PM_{2.5}$ emissions, where low stacks can lead to mostly local health impacts. NO_x emission impacts are also more distributed than primary $PM_{2.5}$impacts and show larger dependence on local weather conditions, as cold temperatures favor the formation of secondary $PM_{2.5}$ due to NO_x by shifting the chemical equilibrium of NH_4NO_3, while high ozone levels and high solar radiation speed up the conversion of NO_x. The NH_3 health impacts covered by the present study are largely linked to NH_3 emissions from NO_x abatement, but these impacts are generally substantially lower than for the other three substances causing $PM_{2.5}$ health impacts (42, 43).

Extended limitations and outlook. The main limitations of our study and options for improvement include:

- Complex setups of power plants, for example including common steam mains, several boilers that are connected to one generator or vice versa, combined cycle power plants of all types, co-generation power plants with heat supply at several different thermodynamic conditions, auxiliary boilers, auxiliary burners or heat integration of power plants with desalination could only be covered in a simplified form by our models. In particular, industrial applications of heat and power have very specific requirements and thus also result in unique and highly site-specific energy supply systems. Acquiring the data for an exhaustive coverage will probably be impossible but a deeper look into specific sectors (e.g. the chemical industry, minerals and mining, pulp and paper, etc.) may allow to improve the existing models with appropriate default assumptions.
- Missing emission measurement data for some important country/ pollutant combinations remains a key limitation of the present work. While missing $PM_{2.5}$ data in Europe is deeply regrettable, it may to a certain degree be appropriate to estimate it from PM_{10} emissions as we did here. More critical are the data gaps in developing and least developed countries, such as India, Indonesia, Brazil, Nigeria, and others but also in Russia, as emission records as well as information about the involved technologies are limited. Thus, the emission estimates in such regions rely on weaker assumptions than in most developed regions, while also the emission estimation techniques are based on developed countries. This gap could potentially be filled by individual company records, which would involve a large amount of manual work. An alternative could also be emission estimates from satellite remote sensing (which

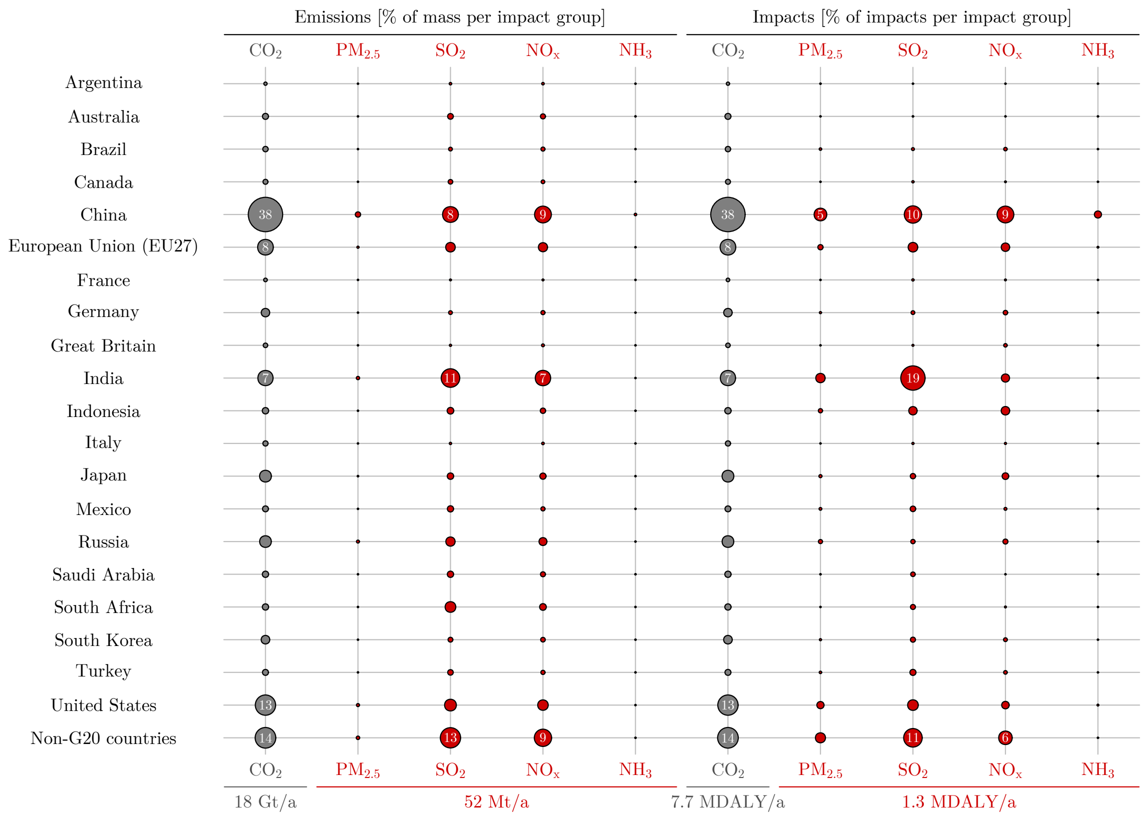

Supplementary figure 5. *Country-level emission and impact comparison in [%] contribution to totals in each category (global warming-related in gray or PM human health impact-related in red). Total amounts for emissions and impacts given at the bottom of the figure, but impacts between the global warming and PM are difficult to compare due to methodological differences. The split of health impacts between $PM_{2.5}$, SO_2, NO_x and NH_3 is 18 %, 53 %, 27 % and 2 %, respectively. The full data is presented in supplementary table 25.*

have already been used for flaring). The usefulness has been checked briefly as part of the current work already, but a challenge were the common emission clusters of several facilities located close to each other, so the attribution of total emissions to individual emission sources would require a substantial amount of additional work.

- While location data was generally of high quality globally, better locations for small-scale power generation and Chinese cement plants would be desirable since especially $PM_{2.5}$ emissions from low stacks may have massive local health impacts. For cement plants, this may be possible as they are relatively large facilities and can be located from satellite imagery within reasonable amounts of time (which has been done for the larger Chinese cement plants as part of this work), but unclear naming and several cement plants in one area would pose some problems. For small-scale power generation, a recurring update of locations via (44) would probably be useful as the data in there is continuously expanded but there are inherent limits to this when the names of the unit correspond to a company that has changed names or where they are not descriptive. In addition, there may also be a gap of small units in (1) for some countries.
- So far, the emission modeling in this work follows a gate-to-gate approach, which only covers on-site emissions but excludes upstream or downstream emissions. This is problematic where related activities may happen at the main site or elsewhere, as this limits the comparability of sites with each other. For example, it is common for many Chinese steel mills to operate their own coke ovens on site, while in other regions of the world, this is less frequently the case. Also, some iron pellet plants are located at the steel mills, while more frequently, they are found close to iron ore mining sites. Thus, the inclusion of steel mill supply chains - and also other supply chains - would help to improve the global comparability of sites. In addition, the data from this work would then be ready for integration into the ecoinvent life cycle inventory database (35), where its high level of regional detail could help to reduce uncertainties of core data drastically.
- The choice of the PM fate and effect model in the present work may somewhat shape its outcomes as the calculation of marginal, average or linear effects from pollutant concentration changes implicitly leads to some regional weighting with the general trends of that having been discussed in (32). The calculation in there has been made for ground-level emissions and a cut-off radius of 250 km from the emission source, while here the emissions can be from any height and the radius has been set to 1000 km. Thus, the overall influence of the marginal, average or linear effect model might be different. Nevertheless, the general trends will remain the same as the change from marginal to average or linear effect estimates will increase the assessed health impact reduction potential in parts of Asia (mostly China and India), which were also identified as biggest contributors with a marginal effect model.

The overall results and conclusions will therefore remain unchanged.

- While the human health impacts from PM are observed and statistically analyzed from current health and exposure statistics, the health impacts of climate change are modeled for a time horizon of 100 a and are in consequence substantially more uncertain (45). On the other hand, the quantification of CO_2 emissions from industrial activities in the absence of abatement measures is possible from carbon mass balances, while the emissions of primary $PM_{2.5}$ and the precursors of secondary $PM_{2.5}$ are also subject to operational practices that are globally not always documented, which leads to higher uncertainties for PM-related emissions in comparison to CO_2. And then, climate change poses the risk of causing malnutrition and increasing cases of diseases such as diarrhea or malaria (45), while PM air pollution is found responsible for lower respiratory infections, ischaemic heart disease, lung cancer, chronic obstructive pulmonary disease and stroke. These different types of human health impacts are all inherently difficult to compare.
- The present work only investigates the total annual emissions and health impacts, while a strong seasonality of environmental conditions in combination with a seasonality in emission patterns could lead to underestimation or overestimation of the related impacts (32). Thus, an increased temporal resolution of emission patterns (for example in case of co-generation power plants that operate differently in summer and winter) might be worth investigating in terms of its influence on the overall results.
- Stack heights have an important influence on the distribution of pollutants but for some countries, only very limited actual measurements of stack heights were available. A review of global construction and design guidelines could help to reduce the influence of this type of uncertainty on the overall results and even be integrated with better wind direction and wind speed data than originally used by the model of (32) to provide more realistic pollution patterns.
- The PM health impact assessment currently remains incomplete as there may be more impact pathways that have not been covered by existing estimation methods, there are other pollutants that contribute to PM formation besides primary $PM_{2.5}$, SO_2, NO_x and NH_3 (such as volatile organic compounds) and the causes for PM toxicity impact are not yet well understood (e.g. in terms of toxicity differences between different types of PM). Thus, further development in terms of health impact quantification would be beneficial, which could then be used for a re-assessment of the global reduction potentials.
- While the health impacts of GHG releases and PM are investigated as part of this work, it was also found that heavy metals such as mercury from coal power plants (7) may be significant contributors to total health impacts caused by industrial site. These have not been covered in this study. In addition, the inclusion of upstream supply chains may to some degree have an impact on the comparison of different types of sites, as these have been neglected as well. Oil and gas extraction, processing and transportation may be responsible for substantial releases of GHG emissions (38).
- We observed that the health impact reduction potentials due to PM and CO_2 are difficult to compare despite their shared unit of measure. While this will remain a problem because of the different temporal dimensions that are involved, this may become easier by methodological alignment of their calculation procedures (e.g. in terms of health effect model).

Abbreviations and function variables. Abbreviations used in this paper are shown in supplementary table 1, while function variables are shown in supplementary table 12.

Supplementary bibliography

1. S&P Global. World Electric Power Plants Database (WEPP), version July 2020, 2020.
2. CE Raptis and S Pfister. Global freshwater thermal emissions from steam-electric power plants with once-through cooling systems. *Energy*, 97:46–57, 2016.
3. CE Raptis, C Oberschelp, and S Pfister. The greenhouse gas emissions, water consumption, and heat emissions of global steam-electric power production: a generating unit level analysis and database. *Environ Res Lett*, 15(10), 2020.
4. SE Fick and RJ Hijmans. WorldClim 2: new 1-km spatial resolution climate surfaces for global land areas. *Int J Clim*, 37(12):4302–4315, 2017.
5. S Pfister and S Suh. Environmental impacts of thermal emissions to freshwater: spatially explicit fate and effect modeling for life cycle assessment and water footprinting. *Int J LCA*, 20(7):927–936, 2015.
6. U.S. Energy Information Administration (EIA). Form EIA-860 detailed data 2016, 2019. https://www.eia.gov/electricity/data/eia860/. Accessed on 2019-04-08.
7. C Oberschelp, S Pfister, CE Raptis, and S Hellweg. Global emission hotspots of coal power generation. *Nature Sustainability*, 2(2):113, 2019.
8. International Energy Agency (IEA). IEA Extended World Energy Balances 2018. Paris, 2018. https://doi.org/10.1787/data-00513-en. Accessed on 2018-12-03.
9. China Electricity Council. 2015 National Thermal Power Unit Competition 100MW unit basic information table (in Chinese), 2016. http://kjfw.cec.org.cn.
10. China Electricity Council. 2015 National Thermal Power Unit Competition 300MW unit basic information table (in Chinese), 2016. http://kjfw.cec.org.cn.
11. China Electricity Council. 2016 National Thermal Power Unit Competition 600MW (including 1000MW) unit basic information table (in Chinese), 2017. http://kjfw.cec.org.cn.
12. Central Electricity Authority (CEA). CO2 baseline database for the Indian power sector, v15.0, 2020. http://cea.nic.in. Accessed 2020-06-12.
13. The International Association for the Properties of Water and Steam (IAPWS). IAPWS R4-95(2014) - Revised Release on the IAPWS Formulation 1995 for the Thermodynamic Properties of Ordinary Water Substance for General and Scientific Use, 2014. http://www.iapws.org/relguide/IAPWS-95.html.
14. U.S. Environmental Protection Agency (EPA) - Combined Heat and Power Partnership. Catalog of CHP Technologies, 2017. https://www.epa.gov/chp/catalog-chp-technologies.
15. Blair, N and DiOrio, N and Freeman, J and Gilman, P and Janzou, S and Neises, T and Wagner, M. NREL System Advisor Model (SAM) - Version 2017.9.5, 2017. https://www.nrel.gov/docs/fy18osti/70414.pdf.
16. Wärtsilä. Combustion Engine vs. Gas Turbine - Part Load Efficiency and Flexibility, 2019. https://www.wartsila.com/energy/learn-more/technical-comparisons/combustion-engine-vs-gas-turbine-part-load-efficiency-and-flexibility. Accessed on 2019-12-22.
17. FJ Brooks. GE Gas Turbine Performance Characteristics, 2000. https://www.ge.com/content/dam/gepower-pgdp/global/en_US/documents/technical/ger/ger-3567h-ge-gas-turbine-performance-characteristics.pdf.
18. CL Mutel and S Hellweg. Regionalized life cycle assessment: computational methodology and application to inventory databases. *Environ Sci Technol*, 43(15):5797–5803, 2009.
19. CL Mutel, S Pfister, and S Hellweg. GIS-based regionalized life cycle assessment: how big is small enough? Methodology and case study of electricity generation. *Environ Sci Technol*, 46(2):1096–1103, 2012.
20. Y Yang and R Heijungs. A generalized computational structure for regional life-cycle assessment. *Int J LCA*, 22(2):213–221, 2017.
21. European Environment Agency (EEA). EMEP/EEA air pollutant emission inventory guidebook 2016, 2016. https://www.eea.europa.eu/publications/emep-eea-guidebook-2016.
22. P Kumari, AK Singh, DA Wood, and B Hazra. Predictions of gross calorific value of Indian coals from their moisture and ash content. *J Geol Soc India*, 93(4):437–442, 2019.
23. U.S. Energy Information Administration (EIA). Glossary - Coal - Low-sulfur coal, 2020. https://www.eia.gov/tools/glossary/?id=coal. Accessed on 2020-07-15.
24. United Nations Environment Programme (UNEP). The Global Sulphur Progress Tracker, 2020. https://www.unenvironment.org/resources/toolkits-manuals-and-guides/global-sulphur-progress-tracker. Accessed on 2020-05-31.
25. International Monetary Fund (IMF). World Economic and Financial Surveys - World Economic Outlook - Database - WEO Groups and Aggregates Information, 2020. https://www.imf.org/external/pubs/ft/weo/2020/01/weodata/groups.htm.
26. United Nations Office of the High Representative for the Least Developed Countries, Landlocked Developing Countries and the Small Island Developing States (UN-OHRLLS). List of Least Developed Countries As of 13 December 2018, 2018. http://unohrlls.org/custom-content/uploads/2018/12/list-of-least-developed-countries-rev1.pdf. Accessed on 2020-05-31.
27. Platts. World Electric Power Plants Database (WEPP), version March 2012, 2012.

28. U.S. Environmental Protection Agency (EPA). Air Emissions Factors and Quantification - AP 42, Fifth Edition Compilation of Air Pollutant Emissions Factors, Volume 1 - Stationary Point and Area Sources, 2011. https://www.epa.gov/air-emissions-factors-and-quantification/ap-42-compilation-air-emissions-factors#5thed.
29. U.S. Environmental Protection Agency (EPA). CATC Air Pollution Technology Fact Sheets (FS) and Technical Bulletins (TB), 2003. https://www.epa.gov/catc/clean-air-technology-center-products#factsheets. Accessed on 2020-09-25.
30. T Lecomte, J Ferreria de la Fuente, F Neuwahl, M Canova, A Pinasseau, I Jankov, T Brinkmann, S Roudier, and L Delgado Sancho. Best Available Techniques (BAT) Reference Document for Large Combustion Plants, 2017. http://dx.doi.org/10.2760/949.
31. M Lackner, A Palotás, and F Winter. *Combustion: from basics to applications*. John Wiley & Sons, 2013.
32. C Oberschelp, S Pfister, and S Hellweg. Globally regionalized monthly life cycle impact assessment of particulate matter. *Environ Sci Technol*, 54(24):16028–16038, 2020.
33. Intergovernmental Panel on Climate Change (IPCC). 2006 IPCC Guidelines for National Greenhouse Gas Inventories - Volume 2 - Energy, 2006. https://www.ipcc-nggip.iges.or.jp/public/2006gl/vol2.html.
34. U.S. Environmental Protection Agency (EPA). Air Emissions Inventories - 2017 National Emissions Inventory (NEI) Data, 2020. https://www.epa.gov/air-emissions-inventories/2017-national-emissions-inventory-nei-data. Accessed on 2020-06-08.
35. Ecoinvent centre. Ecoinvent v3.7 database - cut-off system model. Zurich, Switzerland, 2020. http://www.ecoinvent.org/.
36. SA Miller and FC Moore. Climate and health damages from global concrete production. *Nature Climate Change*, 10:439–443, 2020.
37. L Cabernard and S Pfister. A highly resolved MRIO database for analyzing environmental footprints and Green Economy Progress. *Sci Tot Env*, 755(1):142587, 2021.
38. United Nations Framework Convention on Climate Change (UNFCCC). Greenhouse Gas Inventory Data - Detailed data by Party. New York City, NY, US, 2020. http://di.unfccc.int/detailed_data_by_party. Accessed on 2020-12-06.
39. M Crippa, G Oreggioni, D Guizzardi, M Muntean, E Schaaf, E Lo Vullo, E Solazzo, F Monforti-Ferrario, JGJ Olivier, and E Vignati. Fossil CO2 and GHG emissions of all world countries - 2019 Report, 2019. https://dx.doi.org/10.2760/687800.
40. M Crippa, E Solazzo, G Huang, D Guizzardi, E Koffi, M Muntean, C Schieberle, R Friedrich, and G Janssens-Maenhout. High resolution temporal profiles in the Emissions Database for Global Atmospheric Research. *Nature Scientific Data*, 7(121):1–17, 2020.
41. GBD 2019 Diseases and Injuries Collaborators. Global burden of 369 diseases and injuries in 204 countries and territories, 1990–2019: a systematic analysis for the Global Burden of Disease Study 2019. *The Lancet*, 396(10258):1204–1222, October 2020.
42. The ecoinvent Association. Ecoinvent geography definitions v2.3, 2019. Available online at https://geography.ecoinvent.org/#data-formats. Accessed on 2019-08-07.
43. European Environment Agency (EEA). European Union emission inventory report 1990-2018 under the UNECE Convention on Long-range Transboundary Air Pollution (LRTAP), 2020. https://www.eea.europa.eu/ds_resolveuid/c48fe5a189e5484095dcb509da927a36.
44. Google LLC. Google Geocoding API. Mountain View, CA, US, 2020. https://maps.googleapis.com. Accessed several times throughout 2020.
45. F Verones, S Hellweg, A Antón, LB Azevedo, A Chaudhary, N Cosme, S Cucurachi, L de Baan, Y Dong, P Fantke, L Golsteijn, M Hauschild, R Heijungs, O Jolliet, R Juraske, H Larsen, A Laurent, CL Mutel, M Margni, M Nunez, M Owsianiak, S Pfister, T Ponsioen, P Preiss, RK Rosenbaum, PO Roy, S Sala, Z Steinmann, R van Zelm, R van Dingenen, M Vieira, and MAJ Huijbregts. LC-IMPACT: A regionalized life cycle damage assessment method. *J Ind Ecol*, 24:1201–1219, 2020.
